# Cholangiogenic potential of human deciduous pulp stem cell-converted hepatocyte-like cells

**DOI:** 10.1186/s13287-020-02113-8

**Published:** 2021-01-13

**Authors:** Ratih Yuniartha, Takayoshi Yamaza, Soichiro Sonoda, Koichiro Yoshimaru, Toshiharu Matsuura, Haruyoshi Yamaza, Yoshinao Oda, Shouichi Ohga, Tomoaki Taguchi

**Affiliations:** 1 Department of Pediatric Surgery, Kyushu University Graduate School of Medical Sciences, Fukuoka, Japan; 2 Department of Anatomy, Faculty of Medicine, Public Health and Nursing, Universitas Gadjah Mada, Yogyakarta, Indonesia; 3 Department of Molecular Cell Biology and Oral Anatomy, Kyushu University Graduate School of Dental Science, 3-1-1 Maidashi, Higashi-ku, Fukuoka, 812-8582 Japan; 4 Department of Pediatric Dentistry, Kyushu University Graduate School of Dental Science, Fukuoka, Japan; 5 Department of Anatomic Pathology, Kyushu University Graduate School of Medical Sciences, Fukuoka, Japan; 6 Department of Pediatrics, Kyushu University Graduate School of Medical Sciences, Fukuoka, Japan; 7 Fukuoka College of Health Sciences, Fukuoka, Japan

**Keywords:** Human deciduous pulp stem cell-converted hepatocyte-like cells, Intrahepatic bile duct regeneration, Cholangiocyte, Tumor necrosis factor alpha, Chronic liver fibrosis

## Abstract

**Background:**

Stem cells from human exfoliated deciduous teeth (SHED) have been reported to show the in vivo and in vitro hepatic differentiation, SHED-Heps; however, the cholangiogenic potency of SHED-Heps remains unclear. Here, we hypothesized that SHED-Heps contribute to the regeneration of intrahepatic bile duct system in chronic fibrotic liver.

**Methods:**

SHED were induced into SHED-Heps under cytokine stimulation. SHED-Heps were intrasplenically transplanted into chronically CCl_4_-treated liver fibrosis model mice, followed by the analysis of donor integration and hepatobiliary metabolism in vivo. Immunohistochemical assay was examined for the regeneration of intrahepatic bile duct system in the recipient liver. Furthermore, SHED-Heps were induced under the stimulation of tumor necrosis factor alpha (TNFA).

**Results:**

The intrasplenic transplantation of SHED-Heps into CCl_4_-treated mice showed that donor SHED-Heps behaved as human hepatocyte paraffin 1- and human albumin-expressing hepatocyte-like cells in situ and ameliorated CCl_4_-induced liver fibrosis. Of interest, the integrated SHED-Heps not only expressed biliary canaliculi ATP-binding cassette transporters including ABCB1, ABCB11, and ABCC2, but also recruited human keratin 19- (KRT19-) and KRT17-positive cells, which are considered donor-derived cholangiocytes, regenerating the intrahepatic bile duct system in the recipient liver. Furthermore, the stimulation of TNFA induced SHED-Heps into KRT7- and SRY-box 9-positive cells.

**Conclusions:**

Collectively, our findings demonstrate that infused SHED-Heps showed cholangiogenic ability under the stimulation of TNFA in CCl_4_-damaged livers, resulting in the regeneration of biliary canaliculi and interlobular bile ducts in chronic fibrotic liver. Thus, the present findings suggest that SHED-Heps may be a novel source for the treatment of cholangiopathy.

**Supplementary Information:**

The online version contains supplementary material available at 10.1186/s13287-020-02113-8.

## Background

Orthotopic liver transplantation (OLT) is the only option to ameliorate liver refractory diseases, such as chronic liver fibrosis [1]. However, approximately 10% of patients with end-staged hepatic disorders are not able to receive the benefit owing to the extended waiting period and the shortage of donor organs [2, 3]. Human primary hepatocyte transplantation (hPHepT) becomes a considerable alternative to OLT, especially in liver-based inborn errors of hepatic metabolism and acute/fulminant liver failure [4–6]. Despite the clinical advantages of hPHepT, such as a less-invasive procedure, a current clinical limitation of clinical hPHepT is set by the shortage of effective donor hepatocytes and temporal efficacy [7].

Diverse human mesenchymal stem/stromal cell (MSC)-derived hepatocyte-like cells (HLCs) have been investigated as alternatives to hPHeps [8, 9]. Human deciduous pulp stem cells were first identified in the dental pulp tissues of exfoliated deciduous teeth, namely stem cells from human exfoliated deciduous teeth (SHED) [10], and are beneficial for the treatment of autoimmune, liver, and spinal injury diseases [11–13]. Recent studies demonstrated that SHED exhibit a hepatic potency under a sequential stimulation of hepatogenic cytokines, as referred to SHED-Heps, but SHED-Heps were immature and showed limited-function as hepatocytes [14, 15].

Chronic injury often causes ductular reaction in liver [16, 17]. Liver fibrosis is closely associated with the ductular reaction, resulting in bile excretion damage [18]. However, which therapeutic option regenerates intrahepatic bile ducts in cholestasis associated with liver diseases has yet not been elucidated. In this study, we investigated whether donor SHED-Heps recruited intrahepatic bile duct system, as well as reconstructed parenchymal hepatocytes, in fibrotic liver of chronically CCl_4_-treated mice. Further, we also investigated if SHED-Heps could be induced into cholangiocyte marker-expressing cells cultured under tumor necrosis factor alpha (TNFA) stimulation. Thus, this study aims to demonstrate our hypothesis of the cholangiogenic potency in vivo of SHED-Heps.

## Methods

### Mice

C57BL/6J mice (female, 6-week old) were purchased from Charles River Laboratories Japan (Yokohama, Japan). All animal experiments were approved by the Institutional Animal Care and Use Committee of Kyushu University (approval no. A21-044-1 and A20-041-0). All animals were housed in temperature- and light-controlled conditions with a 12-h light and dark cycle, and fed water and standard pellet chow ad libitum.

### Antibodies

Additional file 1 Supplementary Table 1 lists the antibodies used in this study.

### Isolating, culturing, and characterizing SHED and generating SHED-Heps

SHED were isolated, cultured, and characterized according to previous studies [10, 11] (Additional file 1: Supplementary Fig. 1). The details of SHED culture procedures are as described in the Additional file 1: Supplementary Methods.

### Induction of SHED-Heps

P3 SHED were seeded at 2.5 × 10^5^ cells per dish on human fibronectin-coated 100-mm culture dishes (Corning) and maintained in a growth medium. After they reached confluency, SHED were treated with epidermal growth factor (EGF; 20 ng/mL; PeproTech, Rocky Hill, NJ) and fibroblast growth factor 2 (FGF2; 10 ng/mL; PeproTech) in Iscove’s modified Dulbecco’s media (IMDM; Thermo Fisher Scientific, Waltham, MA) and premixed antibiotics of 100 U/ml penicillin and 100 μg/ml streptomycin (premixed P/S antibiotics; Nacalai Tesque, Kyoto, Japan) for 2 days [14, 19] (Supplementary Fig. S2a). Subsequently, the cells were cultured using hepatogenic cytokines and regents. Initially, the cells were stimulated with FGF2 (10 ng/mL; PeproTech), hepatocyte growth factor (HGF; 20 ng/mL; PeproTech), and nicotinamide (0.61 g/L; Merck, Darmstadt, Germany) in IMDM (Thermo Fisher Scientific) and premixed P/S antibiotics (Nacalai Tesque) for 7 days. Finally, they were stimulated with oncostatin M (20 ng/mL; PeproTech), dexamethasone (1 μM; Merck), insulin-transferrin-selenium premix solution (ITS) premix (1×; Corning), and premixed P/S antibiotics (Nacalai Tesque) for 21 days. Each medium was changed twice weekly. The generated SHED-Heps were re-seeded at 0.1 × 10^6^ per well in low cell attachment PrimeSurface 96 U multiwell plates (Sumitomo Bakelite, Tokyo, Japan). The medium contained oncostatin M (20 ng/mL; Pepro Tech), dexamethasone (1 mM; Merck), ITS premix (1×; Thermo Fisher Scientific), and premixed P/S antibiotics (Nacalai Tesque) in IMDM (Thermo Fisher Scientific) for 7 days, as described previously [15].

### Transplantation of SHED-Heps (SHED-HepT) into CCl_4_-treated chronic liver fibrosis model mice

A CCl_4_ solution in olive oil (1.0 mL/kg body weight; CCl_4_ to olive oil = 1:4 volume/volume; Wako Pure Chemicals, Osaka, Japan) was intraperitoneally injected into C57BL/6J mice twice a week for 8 weeks, as described previously [12, 19]. Age-matched C57BL/6J mice infused with olive oil (Wako Pure Chemicals) were used as controls. The generated SHED-Heps were washed with sterilized with phosphate-buffered saline and kept in cold phosphate-buffered saline (PBS) on ice and quickly infused into the recipient spleen within 10 min after the preparation to maintain the initially prepared donor cell viability. Four-week-CCl_4_-treated C57BL/6J mice were intrasplenically infused SHED-Heps (1 × 10^6^/10 g body weight in 100 μL of PBS). Age-matched 4-week-CCl_4_-treated C57BL/6J mice were infused with PBS (100 μL) as experimental controls. All mice did not receive any immunosuppressants throughout the experiment.

### In vivo monitoring of transplanted donor cells

SHED-Heps were labeled with XenoLight DiR NIR Fluorescent Dye (DiR; 10 μg/mL; Perkin Elmer, Waltham, MA) for 30 min at 37 °C. The DiR-labeled cells or non-labeled SHED-Heps (each 1 × 10^6^ in 100 μL of PBS) were intrasplenically infused into 4-week-CCl_4_-treated C57BL/6J mice. Ventral images of the mice were obtained 24 h after infusion with an optical in vivo imaging system IVIS Lumina III (Perkin Elmer) using living image software (Perkin Elmer).

### Isolation of whole liver cells (WLCs) from recipient CCl_4_-treated mice

Mouse livers were perfused with Liver Perfusion Medium (Thermo Fisher Scientific) at 37 °C for 5 min through the vena cava, followed by 0.1% collagenase type I (Worthington Biochemicals, Lakewood, NJ) at 37 °C for 15 min. The liver samples were gently loosened, and WLCs were obtained through a 70-μm cell strainer.

### Histological assays of mouse liver tissues

Tissue samples were fixed with formalin and embedded in paraffin. The paraffin sections were used for immunohistochemical and double immunofluorescent analyses, as described in the Additional file 1: Supplementary Methods, previously [11, 14]. The primary antibodies used in immunohistochemical and immunofluorescent assays are summarized in the Additional file 1: Supplementary Table 2. Immunohistochemical and immunofluorescent controls were treated with isotype-matched antibodies instead of primary antibodies. Paraffin sections of normal human and mouse liver tissues were stained with the primary antibodies and examined for cross-reactivity of the primary antibodies. Several paraffin sections were treated with hematoxylin and eosin staining.

### In vivo bile assay

Total bilirubin in mouse serum was measured by colorimetric assay using Bilirubin QuantiChrom Assay Kit (BioAssay Systems, Hayward, CA) according to the manufacturer’s instructions. Human albumin (ALB) in mouse serum was analyzed by enzyme-linked immunosorbent assay (ELISA) using human albumin ELISA Quantitation set (Behtyl Laboratory, Montgomery, TX). Feces were collected after fasting overnight. Bile acid in feces was measured by colorimetric assay using Total Bile Acid-Test Wako (Wako Pure Chemical).

### In vivo biliary secretion assay

C57BL/6J mice were intravenously infused with cholyl-lysyl-fluorescein (CLF; 1 mM, 100 μL; Corning, Corning, NY) and harvested 2 h after injection. The amount of CLF in the liver, serum, and urine was quantitated by measuring fluorescence, as described previously [20].

### In vitro biliary function assay

Gene expression of uridine 5′-diphospho-glucuronosyltransferase 1A1 (*UGT1A1*), *membrane metalloendopeptidase* (*MME*), *ATP-binding cassette transporter B1* (*ABCB1*), *ABC11*, and *ABCC2* was analyzed in SHED-Heps by reverse transcription-quantitative polymerase chain reaction (RT-qPCR), as described in the Additional file 1: Supplementary Methods. Distribution of MME, ABCB1, ABCB11, and ABCC2 in SHED-Hep spheroids was analyzed by immunohistochemistry. To analyze hepatobiliary function assays, SHED-Heps were incubated with indirect bilirubin (25 μM; Merck, Darmstadt, Germany) in Ca^2+^-free HBSS (Nacalai Tesque) at 37 °C for 60 min and used for determining direct bilirubin by colorimetric assay using QuantiChrom Bilirubin Assay Kit (BioAssay Systems) according to the manufacturer’s instructions. SHED-Heps were also incubated with CLF (5 μM; Corning) in Ca^2+^-free Hank’s balanced salt solution (HBSS; Nacalai Tesque) at 37 °C for 15 min. Intact SHED and human intrahepatic biliary epithelial cells (AXOL, Cambridge, UK) were used as controls.

### Immunophenotype analysis of SHED-Heps and WLCs

The expression of CD90, epithelial cell adhesion molecule (EPCAM), promin-1 (PROM1), MME, CD146, and CD34 were analyzed in SHED-Heps and WLCs by flow cytometric (FCM) analysis, as described in the Additional file 1: Supplementary Methods.

### Induction of SHED-Heps into cholangiocyte marker-expressing cells

SHED-Heps were maintained in William’s medium E (Thermo Fisher Scientific) and premixed P/S antibiotics (Nacalai Tasque) supplemented with or without TNFA (20 ng/mL; PeproTech) for 4 days. Gene expression of human hepatocyte *nuclear factor 6* (*HNF6*), human *SRY-box 9* (*SOX9*), human *cytokeratin 7* (*KRT7*), and human *KRT19* was analyzed by RT-qPCR. The distribution of human SOX9, human KRT7, and human ALB was analyzed by immunofluorescence, as described in the Additional file 1: Supplementary Methods.

### Statistical analysis

Statistical results were expressed as means ± standard deviation (SEM) from, at least, triplicate measurements. Comparisons between two groups were analyzed by independent two-tailed Student’s *t* tests. Multiple group comparison was analyzed by one-way repeated measures analysis of variance followed by the Tukey post hoc test. The values of *P* < 0.05 were considered statistically significant. All statistical analyses were performed using PRISM 6 software (GraphPad, Software, La Jolla, CA).

## Results

### SHED are induced to hepatocyte-lineage committed cells in vitro

We isolated SHED with a criteria of MSCs, including attached colony formation, cell surface antigen expression, and multipotency into osteoblasts, chondrocytes, and adipocytes [21] (Additional file 1: Supplementary Fig. 1). We cultured SHED under a hepatogenic condition (Additional file 1: Supplementary Fig. 2a). We found a morphological change of spindle-shaped intact SHED into hexagonal-shaped SHED-Heps and bile canaliculi-like structures intercellular space of SHED-Heps under the hepatogenic condition (Additional file 1: Supplementary Fig. 2b). RT-qPCR showed that SHED-Heps expressed higher levels for *KRT18*, *ALB*, *transthyretin* (*TTR*), *HNF1A*, *HNF4A*, *nuclear receptor subfamily 1 group I member 2* (*NR1I2*), and *peroxisome proliferator activated receptor alpha* (*PPARA*) than intact SHED, but lower in SHED-Heps than in hPHeps; meanwhile, SHED-Heps and intact SHED did not express *alpha fetoprotein* (*AFP*), but hPHeps expressed (Additional file 1: Supplementary Fig. 2c). Immunohistochemical analysis detected ALB in SHED-Heps, but not in SHED (Additional file 1: Supplementary Figs. 2d). SHED-Heps showed higher levels of various hepatocyte-specific genes, which are related to urea cycle, glycogen, amino acid, lipid metabolism, xenobiotics, and production of coagulation factor, than intact SHED, but lower than hPHeps by RT-qPCR (Additional file 1: Supplementary Fig. 3).

SHED-Heps exhibited the increased release of glucose, triglyceride, ALB, and fibrinogen, but not AFP into the CM, the decreased ammonia in the CM, the increased intracellular urea, and the enhanced activity of cytochrome P450 3A4 compared to intact SHED (Additional file 1: Supplementary Figs. 4a–4e). SHED-Heps showed the ability of different metabolites storage including indocyanine green uptake and release after 6 h, cytoplasmic accumulation of acetylated low-density lipoprotein uptake, and glycogen storage, but intact SHED did not exhibit any storage function (Additional file 1: Supplementary Figs. 4f–4i). The functions were lower in SHED-Heps than in hPHeps (Additional file 1: Supplementary Fig. 4), indicating that SHED-Heps were immature and limited-functional hepatic committed cells.

### SHED-HepT ameliorates liver fibrosis in chronically CCl_4_-treated mice

**Fig. 1:**
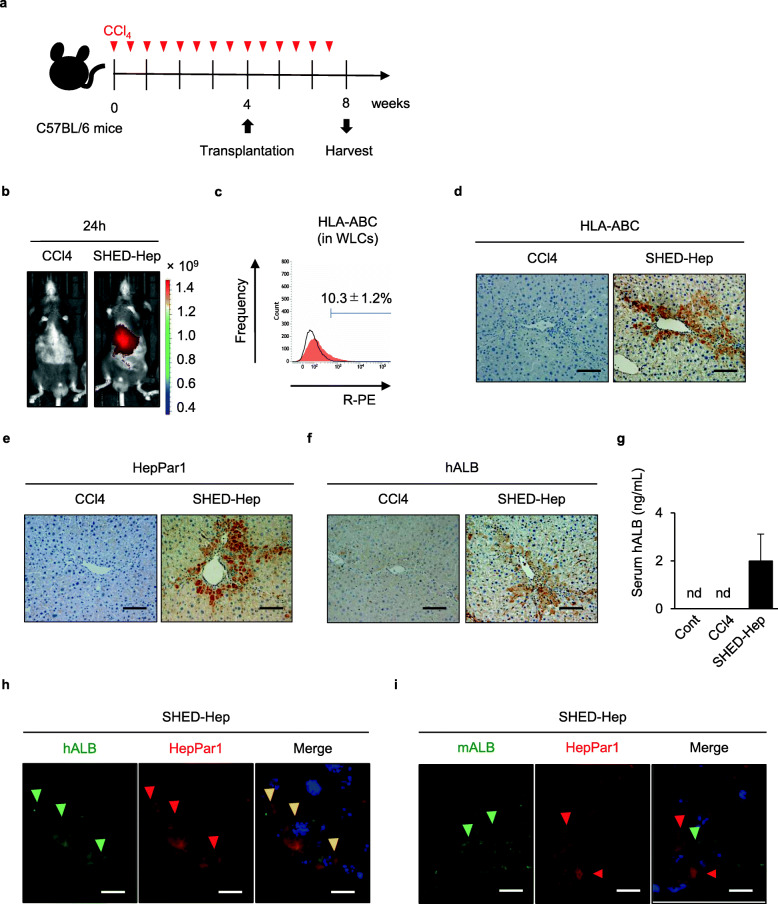
Transplanted donor SHED-Heps engraft without cell fusion in livers of recipient CCl_4_-treated mice. **a** A schema of SHED-Hep transplantation (SHED-HepT) into chronically CCl_4_-treated mice. Mice were intraperitoneally treated with CCl_4_ (1 mg/kg in olive oil, red arrowheads) twice a week for 8 weeks and administrated SHED-Heps (1.0 × 10^6^/mouse) 4 weeks after CCl_4_ treatment. The mice were harvested 8 weeks after CCl_4_ treatment. **b** Representative images of in vivo kinetics of donor SHED-Heps were detected in CCl_4_-treated mice 24 h after SHED-HepT by DiR labeling. **c–i** Distribution of donor SHED-Heps was analyzed in the livers 4 weeks after the transplantation. Representative histogram of human leucocyte antigens A, B, and C (HLA-ABC) expression in the recipient whole liver cells (WLCs) by flow cytometric (FCM) assay. Area filled with red: target antibody-stained histograms; solid line: isotype-matched control-stained histograms. Number indicates averages of the positive rate (**c**). Representative images of HLA-ABC (**d**), hepatocyte paraffin 1 antigen (HepPar1; **e**), and human albumin (hALB; **f**) were detected by immunohistochemical analysis. Serum levels of hALB by enzyme-linked immunosorbent assay (ELISA). *n* = 5. nd, no detection. The graph bars represent the means ± standard error of mean (SEM) (**g**). Representative images of the expression of HepPar1 and hALB (**h**) and HepPar1 and mouse albumin (mALB, **i**) were detected by double immunofluorescent analysis. Nuclei were stained with 4′,6-diamidino-2-phenylindole (DAPI). Merge: merged image. **b, d–i**: Cont, olive oil-treated mice; CCl4, CCl_4_-treated mice; SHED-Hep, SHED-Hep-transplanted CCl_4_-treated mice. **d–f, h, i**: Scale bars, 50 μm (**d**–**f**) and 10 μm (**h, i**)

Four-week-CCl_4_-treated C57BL/6J mice were intrasplenically infused SHED-Heps (1 × 10^6^ per mouse) and subsequently received CCl_4_ for 4 weeks after transplantation (Fig. 1a). Biochemical assays showed the reduced serum levels of aspartate aminotransferase and alanine aminotransferase in recipient mice 4 weeks after transplantation (Additional file 1: Supplementary Fig. 5a). The liver fibrosis was reduced by SHED-HepT, as indicated by the reduced levels of hydroxyproline content, collagen type I mRNA expression, fibrous tissue deposition, and fibrosis score by biochemical analysis, RT-qPCR, Masson Trichrome staining, and Ishak scoring (Additional file 1: Supplementary Figs. 5b–5e). Fibrous rates were 0.39% ± 0.63% (means ± SEM) in the control non-treated liver, 4.29% ± 2.51% in the CCl_4_-treated liver, and 0.91% ± 0.41% in the SHED-Hep-transplanted CCl_4_-treated liver 4 weeks after transplantation by Masson Trichrome staining. SHED-HepT showed the decreased levels of alpha-smooth muscle actin 2, smooth muscle, aorta (ACTA2)-positive cells and fibrogenesis-related marker genes for *Acta2*, *matrix metalloprotease 2*, *transforming growth factor beta*, and *TNFA* in the recipient liver by immunohistochemical analysis and RT-qPCR (Additional file 1: Supplementary Figs. 5f, 5 g).

### Transplanted donor SHED-Heps integrate in liver tissue of chronically CCl_4_-treated mice

In vivo cell tracking analysis showed that fluorescent intensity was detected on the recipient body corresponding to the liver 24 h after DiR-labeled SHED-HepT, but not on control mouse body (Fig. 1b). FCM analysis showed the expression of a ubiquitous human cell marker, human leukocyte antigens A, B, and C (HLA-ABC) on WLCs isolated from the recipient mice 4 weeks after transplantation (Fig. 1c). Immunohistochemical analysis using human-specific antibodies showed that HLA-ABC-, human hepatocyte-specific hepatocyte paraffin 1- (HepPar1-), and human ALB-positive cells were detected in the liver parenchymal periphery of recipient mice (Fig. 1d–f). No signal was detected on human and mouse liver tissues by immunohistochemical control tests using isotype-matched antibodies instead of the human-specific antibodies (Additional file 1: Supplementary Fig. 6). Antibody cross-reactivity test evaluated the human specificity of HLA-ABC, HepPar1, and human ALB antibodies, but not the mouse specificity on human and mouse liver tissues (Additional file 1: Supplementary Figs. 7a–7c). Homing rates of HepPar1-positive area were 3.31% ± 1.63% in SHED-Hep-transplanted group, but not detected in both non-treated and CCl_4_-treated groups. By ELISA, serum human ALB was detected in SHED-Hep-transplanted mice, but not in non-treated and CCl_4_-treated mice (Fig. 1g). By double immunofluorescent analysis, HepPar1-positive cells co-expressed human ALB, but not mouse ALB, in the liver parenchyma of recipient mice (Fig. 1h, i).

### SHED-HepT rescues biliary excretion in chronically CCl_4_-treated mice

**Fig. 2:**
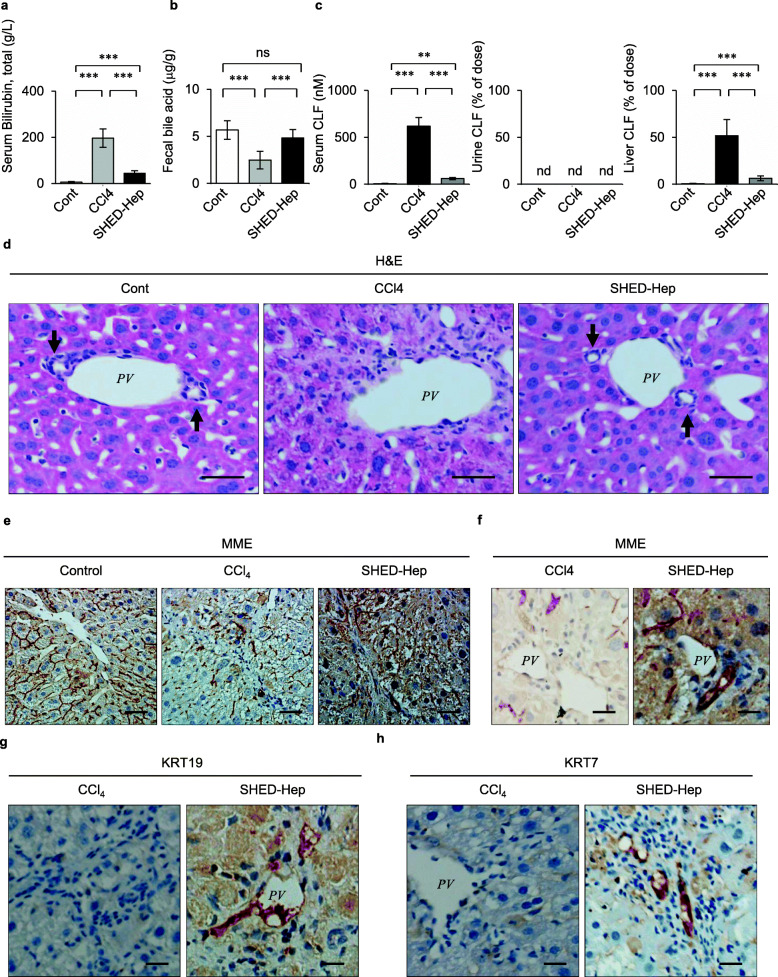
SHED-HepT rescues biliary excretion in recipient CCl_4_-treated mice. **a, b** Levels of serum total bilirubin (**a**) and fecal bile acid (**b**) 4 weeks after transplantation by colorimetry. **c** Cholyl-L-lysyl-fluorescein (CLF) was intravenously infused into mice. The graphs showed the amount of CLF in serum, urine, and liver 2 h after the infusion. **d–h** Liver tissues were analyzed 4 weeks after SHED-HepT. Representative images of the livers were obtained by hematoxylin and eosin (H&E) staining (**d**) and by immunohistochemical analysis using human-specific antibodies to membrane metalloendopeptidase (MME; **e, f**), keratin 19 (KRT19; **g**), and KRT7 (KRT7; **h**). Nuclei were stained with hematoxylin. **a–c** *n* = 5 for all groups. **P < 0.01, ***P < 0.005. nd, no detection; ns, no significance. The graph bars represented the means ± SEM. **d** arrow: bile duct. **d–h** Scale bars, 20 μm (**d, f-h**) and 50 μm (**e**). **d, f–h** *PV*, portal vein

CCl_4_ treatment caused cholestasis in mice, as indicated by the increased levels of serous total bilirubin, the decreased levels of fecal bile acid, and the increased amounts of serous and hepatic CLF by biochemical and biliary excretion analyses (Fig. 2a–c). SHED-HepT recovered the levels of serous total bilirubin and fecal bile acid and the amounts of serous and hepatic CLF 4 weeks after transplantation (Fig. 2a–c). No urinary CLF was found in all experimental groups (Fig. 2c). Histological analysis showed that chronic CCl_4_ treatment damaged biliary canaliculi and interlobular bile ducts in the parenchymal periphery and periportal region of mouse liver; meanwhile, SHED-HepT regenerated the CCl_4_-damaged biliary canaliculi and interlobular bile ducts (Fig. 2d).

MME is a pan marker for bile canaliculi. We then demonstrated that the reactivity of MME was reduced in the liver parenchymal periphery of CCl_4_-treated mice compared to that of control mice by immunohistochemical analysis using anti-human MME antibody (Fig. 2e, Additional file 1: Supplementary Figs. 7d, 8). Interestingly, SHED-HepT increased the MME reactivity in recipient liver (Fig. 2e, Additional file 1: Supplementary Fig. 8). ABCB1, ABCB11, and ABCC2 are bile canaliculi-specific hepatobiliary transporters to excrete bile from hetatocytes into bile canaliculi [22–25]. We showed that ABCB1, ABCB11, and ABCC2 were abundantly expressed in the liver parenchymal periphery of SHED-Hep-transplanted CCl_4_-treated mice compared to that in control CCl_4_-treated mice by immunohistochemical analysis using the corresponding human-specific antibodies (Additional file 1: Supplementary Fig. 9). Immunohistochemical cross-reactivity and control tests determined that the present anti-ABCC2 antibody showed the human specificity; meanwhile, the anti-ABCB1, anti-ABCB11, and anti-MME antibodies did not (Additional file 1: Supplementary Figs. 6, 7d, 9). Double immunofluorescent analysis showed that HepPar1-positive cells co-expressed, at least partially, ABCB1, ABCB11, and ABCC2, as well as MME, in the recipient liver parenchyma (Additional file 1: Supplementary Fig. 10).

Immunohistochemical analysis using anti-human MME antibody showed MME-positive signals in liver interlobular bile ducts of SHED-Hep transplanted mice, but not in those of CCl_4_-treated mice (Fig. 2f). We then performed immunohistochemical analysis using anti-mouse KRT19, anti-human KRT19, and anti-human KRT7 antibodies. Mouse KRT19 immunoreactivity was not detected in the liver tissue of CCl_4_-treated mice; meanwhile, it was found in liver interlobular bile ducts of control mice (Additional file 1: Supplementary Fig. 11a). Human KRT19 and human KRT7 immunoreactivity were detected in liver interlobular bile ducts of SHED-Hep transplanted mice, but not in that of control and CCl_4_-treated mice (Fig. 2g, h, Additional file 1: Supplementary Figs. 11b, 11c). Immunohistochemical cross-reactivity and control tests evaluated human cholangiocyte specificity of anti-human KRT7 and anti-human KRT19 antibodies, as well as mouse cholangiocyte specificity of anti-mouse KRT19 antibody (Additional file 1: Supplementary Fig. 7, 11).

### SHED-Heps exhibit cholangiogenic potency under TNFΑ stimulation in vitro

**Fig.3:**
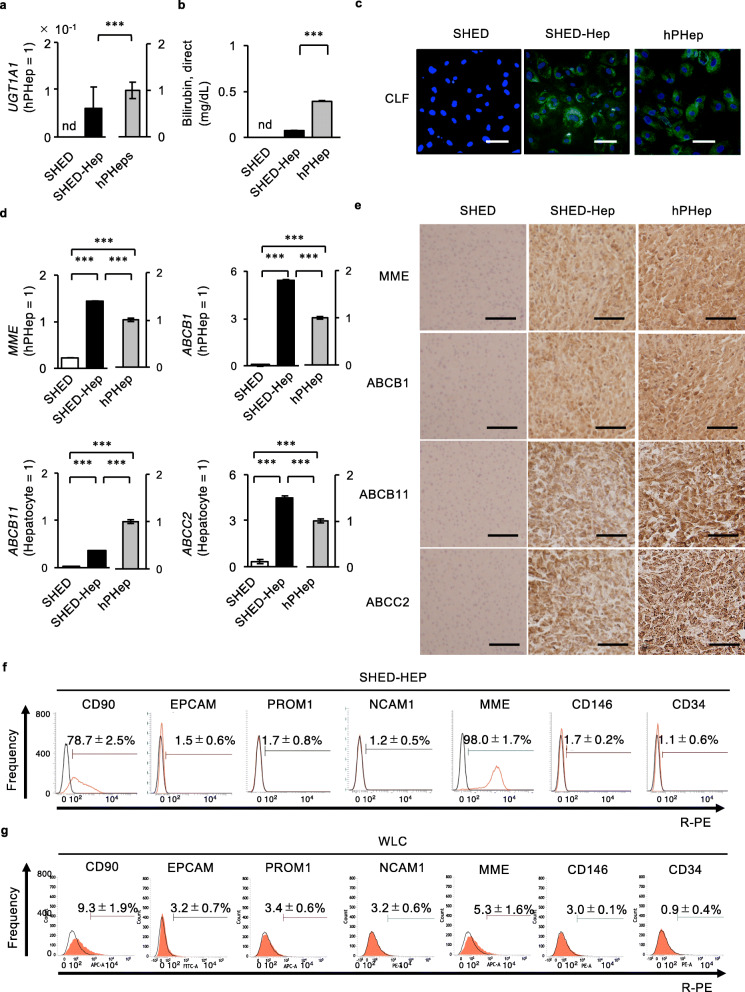
SHED-HepT reconstructs intrahepatic bile ducts in livers of recipient CCl_4_-treated mice. **a–e** In vitro hepatobiliary function was analyzed in SHED-Heps. Gene expression of uridine 5′-diphospho-glucuronosyltransferase 1A1 (*UGT1A1*) by reverse transcription-quantitative polymerase chain reaction (RT-qPCR). Amount of direct bilirubin by colorimetric assay (**b**). Representative images of bile transport were detected by CLF staining. Nuclei were stained with DAPI (**c**). Gene expression of *membrane metalloendopeptidase* (*MME*), *ATP-binding cassette transporter B1* (*ABCB1*), ABCB11, and ABCC2 by RT-qPCR **d**. Representative images of the expression of MME, ABCB1, ABCB11, and ABCC2 were detected by immunohistochemical analysis (**e**). **a–e** hPHep, human primary hepatocyte. **a, b, d** *n* = 5 for all groups. ***P < 0.005. nd, no detection. The graph bars represent the means ± SEM. **a, d** Results are shown as a ratio to hPHeps (hPHep = 1). **c, e** Scale bars, 20 μm. **f, g** Representative histograms of the expression of cell surface markers for hepatocyte progenitors and mesenchymal stem cells in SHED-Heps (**f**) and whole liver cells isolated from recipient mice (WLC) (**g**) were detected by FCM assay. EPCAM: epithelial cell adhesion molecule; MME: membrane metalloendopeptidase; NCAM1: neural cell adhesion molecule; PROM1: promin 1; R-PE: R-phycoerythrin. **f** Red solid line, target antibody-stained histograms; black solid line, isotype-matched control-stained histograms. **g** Area filed with red, target antibody-stained histograms; solid line, isotype-matched control-stained histograms

SHED-Heps expressed *UGT1A1* by RT-qPCR and exhibited the production of direct bilirubin and the function of hepatobiliary transport by colorimetric assay and CLF staining (Fig. 3a–c). RT-qPCR and immunohistochemical analysis demonstrated the gene and protein expression of hepatobiliary transporters, including MME, ABCB1, ABCB11, and ABCC2 in SHED-Heps (Fig. 3d, e). Meanwhile, intact SHED did not show the hepatobiliary ability (Fig. 3a–e). Further FCM analysis revealed that SHED-Heps expressed hepatic markers, CD90 and MME, but did not express critical hepatic progenitor cells (HPCs) markers, EPCAM, PROM1, and neural cell adhesion molecule 1 (NCAM1) [26, 27] (Fig. 3f). SHED-Heps did not also express CD146, which is considered as a critical and primitive marker for MSCs [28], and definitive hematopoietic marker CD34 by FCM analysis (Fig. 3f). Next, we examined the immunophenotype of WLCs isolated from SHED-Hep-transplanted mice by FCM analysis using human-specific antibodies to CD90, MME, EPCAM, PROM1, NCAM1, CD146, and CD34. Of interest, WLCs expressed human EPCAM, human PROM1, human NCAM1, and human CD146, as well as human CD90 and human MME, but not human CD34 (Fig. 3g), implying that the recipient liver environment may induce donor SHED-Heps into cholangiocytes or cholangiocyte-progenitor cells (CPCs).

**Fig. 4:**
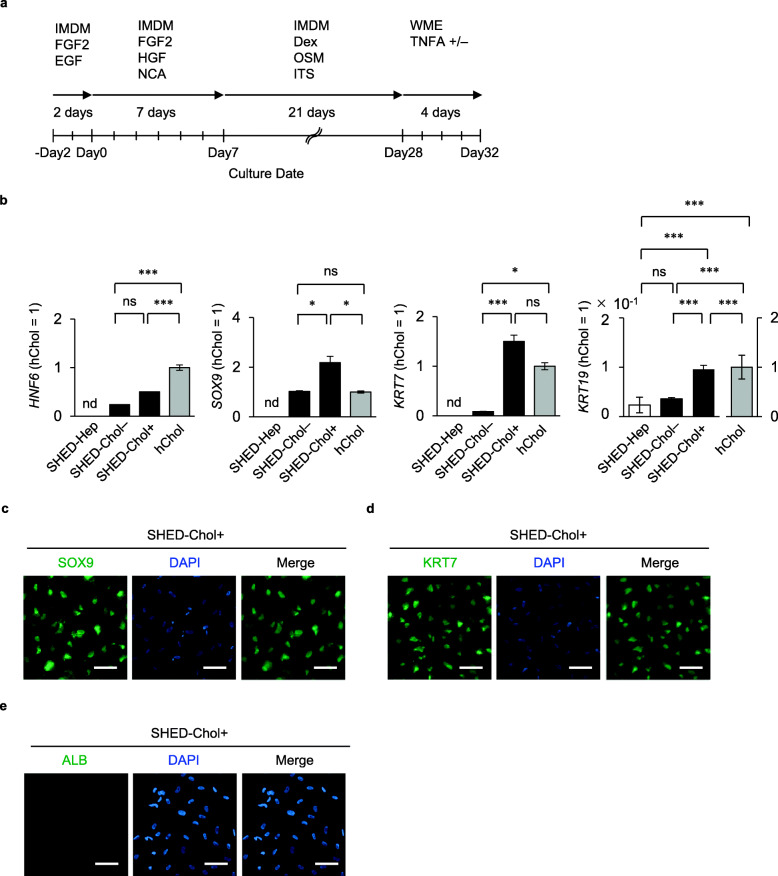
SHED-Heps exhibit a potency into cholangiocyte-like cells. **a** A schema of induction of SHED-Heps into cholangiocyte-like cells (SHED-Chols). Generated SHED-Heps were cultured in William’s medium (WEM) with or without tumor necrosis factor alpha (TNFA +/−) for 4 days. Dex: dexamethasone; EGF: epidermal growth factor; FGF2: fibroblast growth factor 2; HGF: hepatocyte growth factor; IMDM: Iscove’s modified Dulbecco’s medium; ITS: insulin-transferrin-selenium premix solution; NCA: nicotinamide; OSM: oncostatin M. **b** Gene expression of *hepatocyte nuclear factor6* (*HNF6*), *SRY-box 9* (*SOX9*), *KRT7*, and *KRT19* by RT-qPCR analysis. Results are shown as a ratio to human primary cholangiocyte (hChol = 1). *n* = 5 for all groups. *P < 0.05, ***P < 0.005. nd, no detection; ns, no significance. The graph bars represent the means ± SEM. **c–e** Representative images of SOX9, KRT7, and ALB expression were detected by immunofluorescent assay. Nuclei were stained with DAPI. Merge, merged image. Scale bars, 20 μm. **b–e** SHED-Chol−, TNFA-non-stimulated group; SHED-Chol+, TNFA-stimulated group

TNFA can induce hepatocytes into bile ductular cells [29]. The present and previous [12] studies showed that chronic CCl_4_ treatment enhances abundant TNFA expression in mouse liver tissue. SHED-Heps were maintained in William’s E medium stimulated with or without TNFA for 4 days (Fig. 4a). The culture conditions, especially TNFA-co-stimulating condition, enhanced the expression of *HNF6*, *SOX9*, and *KRT7* in SHED-Heps by RT-qPCR (Fig. 4b) and the expression of SOX9 and KRT7, but not human ALB, by immunofluorescent analysis (Fig. 4c–e).

## Discussion

Here, we demonstrated that the donor SHED-Heps integrate into liver parenchyma, in recipient chronically CCl_4_-damaged mice, and regenerate the intrahepatic biliary components. Hepatic damages due to liver fibrosis reduce and lack the distribution of MME in bile canaliculi [30]. Bile canalicular transporters ABCB1, ABCB11, and ABCC2 are defected in congenital cholestasis, including progressive familial intrahepatic cholestasis type2, benign recurrent intrahepatic cholestasis type2, and Dubin Johnson syndrome [31–33]. HPCs are responsible for developing CPCs and cholangiocytes [26]. Hepatocytes exhibit plasticity to recruit bile ductular cells under liver physiological and pathological conditions [34]. Transplanted hepatocytes can differentiate into bile ductular cells in bile duct ligation model animals [35]. A recent study showed that mature hepatocytes undergo the in situ potency of direct differentiation into SOX9-expressing CPCs through HPCs with mesenchymal phenotype [36]. In this study, WLCs from SHED-Hep-transplanted mice contained SHED-derived HPCs and MSCs, but donor SHED-Heps did not contain HPCs and MSCs. The present study showed that integrated SHED-Heps rescued the expression of MME, ABCB1, ABCB11, and ABCC2 in bile canaliculi under chronically CCl_4_-damaged environment. These findings imply a plastic possibility of SHED-Heps to commit to CPCs/cholangiocytes through HPCs in situ and suggest that SHED-Heps might be a source for treating cholestasis associated with bile canaliculi.

Although we revealed that transplanted SHED-Heps recruited bile ductular cells in the recipient livers, the mechanism(s) of SHED-Hep into bile ductular cells remains unknown. Epithelial-mesenchymal transition (EMT) enables mature epithelial cells to undergo transdifferentiation into mesenchymal cells acquired with stem cell-like properties in chronically inflamed tissue [37]. TNFA accelerates EMT via the nuclear factor kappa B–Twist pathway in mammary epithelial cells [38]. Moreover, TNFA can induce the differentiation of hepatocytes into cholangiocytes in organoid culture system [29]. In this study, TNFA enhanced the expression of SOX9 and KRT7 in SHED-Heps. These findings suggest that the inflammatory background by CCl_4_, such as TNFA, may recruit donor SHED-Heps into CPC/cholangiocyte-like cells through HPC state. Further studies will be required to evaluate the molecular mechanisms in TNFA-triggered cholangiocyte-differentiation of SHED-Hep for treating cholangiopathy.

In the present and recent animal studies [12], both systemically transplanted SHED and SHED-Heps recovered chronically CCl4-treated liver fibrosis. Meanwhile, systemically transplanted SHED-Heps, but not SHED, rescued Wilson’s disease disorders [14]. Local implantation of SHED-Hep-aggregates gives a benefit for Factor VIII-lacked Hemophilia A [15]. These findings suggest that diverse approaches using SHED and SHED-Heps give an advantage for treating liver disease. A standard operating procedure (SOP) for manufacturing clinical-grade cell products should be validated before clinical applications [39]. Therefore, currently validated SOP for manufacturing large-scale clinical-grade SHED products [25] might be useful to establish safety-secured and clinical-graded SHED-Hep products. More research will be necessary to improve the quality and safety of SHED-Heps, such as microbial contamination, chromosomal instability, immunogenicity, and tumorigenicity [40]. Post-cryopreserved viability and function of clinical-grade SHED-Hep products should be needed for transplantation [41], because freeze-thawing process reduces the viability and function of donor hepatocytes due to the damage of mitochondrial respiration [42].

In this study, the long-term survival and engraftment of SHED-Heps were not investigated. In a recent study in Wilsons’ disease model animal [14], the long-term survival and engraftment of SHED-Heps are not expected. The reasons why SHED-Heps exhibit the short-term survival and engraftment in the recipient animals are considered due to the immature hepatic differentiation and immunogenicity of donor cells, the xenograft system in immunocompetent animals without any immunosuppressive drug, and the chronical cytotoxic condition after transplantation. On the other hand, SHED-secreting trophic factors including cytokines and extracellular vesicle-containing RNA contents affect immunosuppressive functions [43, 44]. The effect of stanniocalcin 1 secreted from SHED-Heps is anti-hepatitis [14]. Given the results from SHED-Heps transplantation in the intoxicated mice, anti-inflammatory effects by SHED-secreting factors may support the regeneration of bile ducts.

In vivo cell fusion with recipient hepatocyte has been considered as a mechanism to integrate donor cells, such as bone marrow hematopoietic cells and hematopoietic cell-derived hepatocytes, in fumarylacetoacetate hydrolase-deficient immunodeficient and chronic CCl_4_-treated immunocompetent mice [45, 46]. Meanwhile, only bone marrow MSCs can integrated without cell fusion with recipient hepatocytes in the damaged liver [47]. We evaluated that donor SHED can integrate as human HLCs without cell fusion with recipient hepatocytes [12]. Given the present results from fusion failure of donor SHED-Heps, SHED and SHED-Heps may exhibit a unique process to integrate in the recipient damaged liver in vivo. The present hepatic culture system using growth factors and hormones at specific times and in a specific sequence was insufficient to induce SHED into mature and functional HLCs. Based on a recent study, stem cells need to drive them through primitive steak under the stimulation of both inductive and repressive signals for developing into mature and functional hepatocytes [48]. The primitive steak is a critical stage of mimicking developmental biology in an in vitro system, because only the primitive steak can drive definitive endoderm, progenitors, hepatoblasts, and finally mature hepatocytes. Further studies will be necessary to evaluate the in vivo and in vitro mechanism of SHED into mature hepatocytes and cholangiocytes.

## Conclusions

Collectively, SHED-Heps integrate into liver parenchyma, especially its periphery, in recipient chronically CCl_4_-damaged mice and contributes to the regeneration of intrahepatic bile ducts under fibrosis-associated TNFA microenvironment. Thus, SHED-Heps might be a source for treating cholestasis associated with bile canaliculi.

## Supplementary Information

**Additional file 1.** Supplementary Methods. Supplementary References. Supplementary Table 1. The list of specific antibodies used for flow cytometry. Supplementary Table 2. Specific antibodies for immunohistochemistry and immunofluorescence. Supplementary Table 3. List of TaqMan probes for human genes. Supplementary Table 4. List of TaqMan probes for mouse genes. Supplementary Fig. 1. Characterization of stem cells from human exfoliated deciduous teeth (SHED). Supplementary Fig. 2. Hepatogenic properties of SHED. Supplementary Fig. 3. Expression of hepatic function-associated genes in SHED-Heps. Supplementary Fig. 4. Hepatic functions of SHED-Heps. Supplementary Fig. 5. Effects of SHED-Heps transplantation on liver fibrosis in CCl_4_-treated mice. Supplementary Fig. 6. Immunohistochemical control tests. Supplementary Fig. 7. Immunohistochemical specificity of antibodies against human leukocyte antigen A, B, and C (HLA-ABC), human hepatocyte paraffin 1 (HepPar1), human ALB, and human MME. Supplementary Fig. 8. Effects of SHED-Heps transplantation on MME expression in liver of CCl_4_-treated mice. Supplementary Fig. 9. Immunohistochemical localization of biliary transporter markers ATP-binding cassette subfamily B member 1 (ABCB1), ABCB11, and ABCC2. Supplementary Fig. 10. Distribution of biliary canaliculi markers in liver of SHED-Hep-transplanted CCl4-treated mice. Supplementary Fig. 11. Immunohistochemical localization of KRT19 and KRT7.

## Data Availability

All data generated and analyzed during this study are included in this published article and its supplementary information files.

## Abbreviations

ABCB1: ATP-binding cassette transporter B1
ABCB11: ATP-binding cassette transporter B11
ABCC2: ATP-binding cassette transporter C2
AFP: Alpha fetoprotein
ACTA2: Alpha-smooth muscle actin 2, smooth muscle, aorta
ALB: Albumin
CLF: Cholyl-lysyl-fluorescein
CPCs: Cholangiocyte-progenitor cells
EGF: Epidermal growth factor
ELISA: Enzyme-linked immunosorbent assay
EMT: Epithelial-mesenchymal transition
EPCAM: Epithelial cell adhesion molecule
FCM: Flow cytometry
FGF2: Fibroblast growth factor 2
HBSS: Hank’s balanced salt solution
HepPar1: Hepatocyte paraffin 1
HGF: Hepatocyte growth factor
HLA-ABC: Human leukocyte antigens A, B, and C
HLCs: Hepatocyte-like cells
HNF1A: Hepatocyte nuclear factor 1A
HNF6: Hepatocyte nuclear factor 6
HPCs: Hepatic progenitor cells
hPHeps: Human primary hepatocytes
IMDM: Iscove’s modified Dulbecco’s media
ITS: Insulin-transferrin-selenium premix solution
KRT7: Cytokeratin 7
KRT18: Cytokeratin 18
KRT19: Cytokeratin 19
MME: Membrane metalloendopeptidase
MSCs: Mesenchymal stem/stromal cells
NCAM1: Neural cell adhesion molecule 1
NR1I2: Nuclear receptor subfamily 1 group I member 2
PBS: Phosphate-buffered saline
PPARA: Peroxisome proliferator activated receptor alpha
PROM1: Promin-1
RT-qPCR: Quantitative reverse transcription and polymerase chain reaction
SHED: Stem cells from human exfoliated deciduous teeth
SHED-Heps: SHED-derived hepatocyte-like cells
SOP: Standard operating procedure
SOX9: SRY-box 9
TNFA: Tumor necrosis factor alpha
TTR: Transthyretin
UGT1A1: 5′-Diphospho-glucuronosyltransferase 1A1
WLCs: Whole liver cells
