# Supplementary material for: Cholangiogenic potential of human deciduous pulp stem cell-converted hepatocyte-like cells
Source: Stem Cell Res Ther. 2021 Jan 13;12:57. doi: 10.1186/s13287-020-02113-8 (PMC7805240; doi:10.1186/s13287-020-02113-8)
Supplement: Supplementary file 1 — Additional file 1. Supplementary Methods. Supplementary References. Supplementary Table 1. The list of specific antibodies used for flow cytometry. Supplementary Table 2. Specific antibodies for immunohistochemistry and immunofluorescence. Supplementary Table 3. List of TaqMan probes for human genes. Supplementary Table 4. List of TaqMan probes for mouse genes. Supplementary Fig. 1. Characterization of stem cells from human exfoliated deciduous teeth (SHED). Supplementary Fig. 2. Hepatogenic properties of SHED. Supplementary Fig. 3. Expression of hepatic function-associated genes in SHED-Heps. Supplementary Fig. 4. Hepatic functions of SHED-Heps. Supplementary Fig. 5. Effects of SHED-Heps transplantation on liver fibrosis in CCl4-treated mice. Supplementary Fig. 6. Immunohistochemical control tests. Supplementary Fig. 7. Immunohistochemical specificity of antibodies against human leukocyte antigen A, B, and C (HLA-ABC), human hepatocyte paraffin 1 (HepPar1), human ALB, and human MME. Supplementary Fig. 8. Effects of SHED-Heps transplantation on MME expression in liver of CCl4-treated mice. Supplementary Fig. 9. Immunohistochemical localization of biliary transporter markers ATP-binding cassette subfamily B member 1 (ABCB1), ABCB11, and ABCC2. Supplementary Fig. 10. Distribution of biliary canaliculi markers in liver of SHED-Hep-transplanted CCl4-treated mice. Supplementary Fig. 11. Immunohistochemical localization of KRT19 and KRT7. [file 13287_2020_2113_MOESM1_ESM.zip › Ratih(SHEDHepBiliaryNetwork)SupplFiguresSCRT_Revised.pptx]

## Slide 1
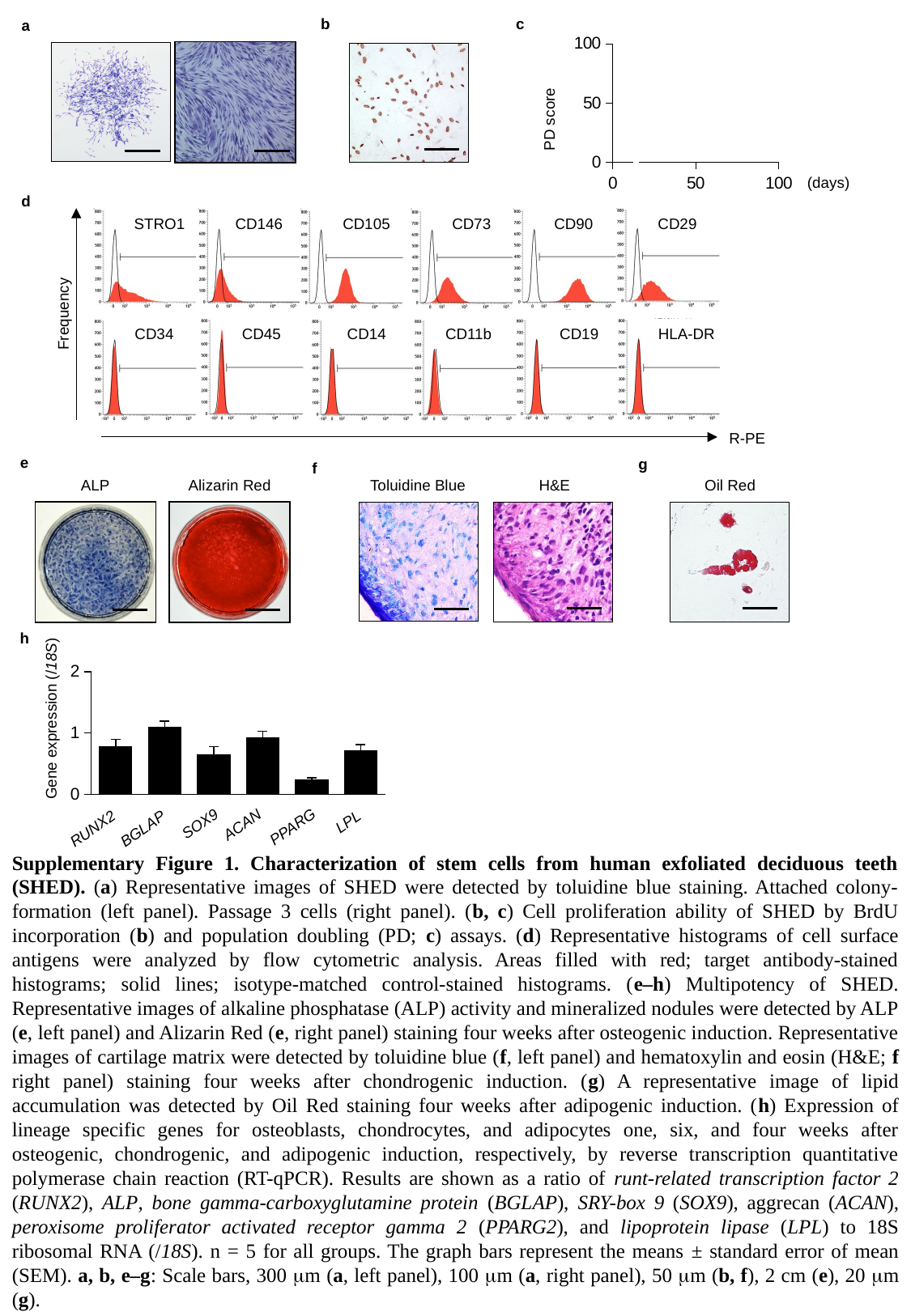

b
c
a
### Chart
| Category | |
|---|---|
PD score
(days)
d
STRO1
CD146
CD105
CD73
CD90
CD29
Frequency
CD34
CD45
CD14
CD11b
CD19
HLA-DR
R-PE
e
g
f
ALP
Alizarin Red
Toluidine Blue
H&E
Oil Red
h
### Chart
| Category | SCAP | SCAP | SCAP | SCAP | SCAP | SCAP |
|---|---|---|---|---|---|---|LPL
SOX9
ACAN
RUNX2
BGLAP
PPARG
Gene expression (/18S)
Supplementary Figure 1. Characterization of stem cells from human exfoliated deciduous teeth (SHED). (a) Representative images of SHED were detected by toluidine blue staining. Attached colony-formation (left panel). Passage 3 cells (right panel). (b, c) Cell proliferation ability of SHED by BrdU incorporation (b) and population doubling (PD; c) assays. (d) Representative histograms of cell surface antigens were analyzed by flow cytometric analysis. Areas filled with red; target antibody-stained histograms; solid lines; isotype-matched control-stained histograms. (e–h) Multipotency of SHED. Representative images of alkaline phosphatase (ALP) activity and mineralized nodules were detected by ALP (e, left panel) and Alizarin Red (e, right panel) staining four weeks after osteogenic induction. Representative images of cartilage matrix were detected by toluidine blue (f, left panel) and hematoxylin and eosin (H&E; f right panel) staining four weeks after chondrogenic induction. (g) A representative image of lipid accumulation was detected by Oil Red staining four weeks after adipogenic induction. (h) Expression of lineage specific genes for osteoblasts, chondrocytes, and adipocytes one, six, and four weeks after osteogenic, chondrogenic, and adipogenic induction, respectively, by reverse transcription quantitative polymerase chain reaction (RT-qPCR). Results are shown as a ratio of runt-related transcription factor 2 (RUNX2), ALP, bone gamma-carboxyglutamine protein (BGLAP), SRY-box 9 (SOX9), aggrecan (ACAN), peroxisome proliferator activated receptor gamma 2 (PPARG2), and lipoprotein lipase (LPL) to 18S ribosomal RNA (/18S). n = 5 for all groups. The graph bars represent the means ± standard error of mean (SEM). a, b, e–g: Scale bars, 300 mm (a, left panel), 100 mm (a, right panel), 50 mm (b, f), 2 cm (e), 20 mm (g).

## Slide 2
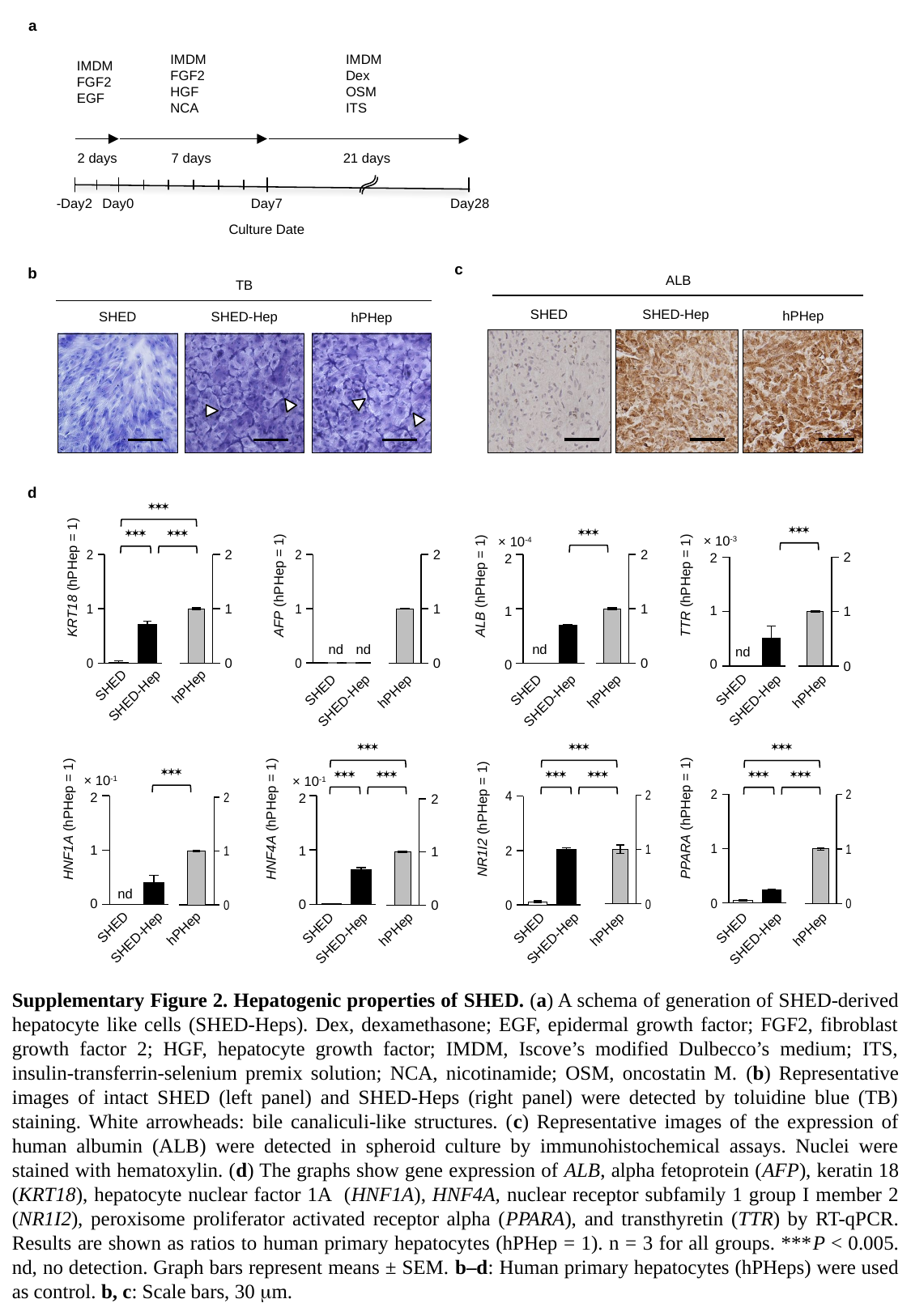

a
IMDM
FGF2
HGF
NCA
IMDM
Dex
OSM
ITS
IMDM
FGF2
EGF
2 days
7 days
21 days
-Day2
Day0
Day7
Day28
Culture Date
c
ALB
SHED
SHED-Hep
hPHep
b
TB
SHED
SHED-Hep
hPHep
d
***
***
***
***
***
× 10-3
× 10-4
2
2
### Chart
| Category | /18s |
|---|---|
| HepG2 | 1.0 |
### Chart
| Category | | |
|---|---|---|
### Chart
| Category | | |
|---|---|---|
### Chart
| Category | /18s |
|---|---|
| HepG2 | 1.0 |
### Chart
| Category | | |
|---|---|---|
### Chart
| Category | /18s |
|---|---|
| HepG2 | 1.0 |
### Chart
| Category | | |
|---|---|---|
### Chart
| Category | /18s |
|---|---|
| HepG2 | 1.0 |KRT18 (hPHep = 1)
AFP (hPHep = 1)
TTR (hPHep = 1)
ALB (hPHep = 1)
1
1
nd
nd
nd
nd
0
0
SHED
hPHep
SHED-Hep
SHED
hPHep
SHED-Hep
SHED
hPHep
SHED-Hep
SHED
hPHep
SHED-Hep
***
***
***
***
***
***
***
***
***
***
× 10-1
× 10-1
2
2
### Chart
| Category | | |
|---|---|---|
### Chart
| Category | |
|---|---|
### Chart
| Category | |
|---|---|
### Chart
| Category | | |
|---|---|---|
### Chart
| Category | | |
|---|---|---|
### Chart
| Category | | |
|---|---|---|
### Chart
| Category | |
|---|---|
### Chart
| Category | /18s |
|---|---|
| HepG2 | 1.0 |PPARA (hPHep = 1)
HNF1A (hPHep = 1)
HNF4A (hPHep = 1)
NR1I2 (hPHep = 1)
1
1
nd
0
0
SHED
hPHep
SHED-Hep
SHED
hPHep
SHED-Hep
SHED
hPHep
SHED-Hep
SHED
hPHep
SHED-Hep
Supplementary Figure 2. Hepatogenic properties of SHED. (a) A schema of generation of SHED-derived hepatocyte like cells (SHED-Heps). Dex, dexamethasone; EGF, epidermal growth factor; FGF2, fibroblast growth factor 2; HGF, hepatocyte growth factor; IMDM, Iscove’s modified Dulbecco’s medium; ITS, insulin-transferrin-selenium premix solution; NCA, nicotinamide; OSM, oncostatin M. (b) Representative images of intact SHED (left panel) and SHED-Heps (right panel) were detected by toluidine blue (TB) staining. White arrowheads: bile canaliculi-like structures. (c) Representative images of the expression of human albumin (ALB) were detected in spheroid culture by immunohistochemical assays. Nuclei were stained with hematoxylin. (d) The graphs show gene expression of ALB, alpha fetoprotein (AFP), keratin 18 (KRT18), hepatocyte nuclear factor 1A (HNF1A), HNF4A, nuclear receptor subfamily 1 group I member 2 (NR1I2), peroxisome proliferator activated receptor alpha (PPARA), and transthyretin (TTR) by RT-qPCR. Results are shown as ratios to human primary hepatocytes (hPHep = 1). n = 3 for all groups. ***P < 0.005. nd, no detection. Graph bars represent means ± SEM. b–d: Human primary hepatocytes (hPHeps) were used as control. b, c: Scale bars, 30 mm.

## Slide 3
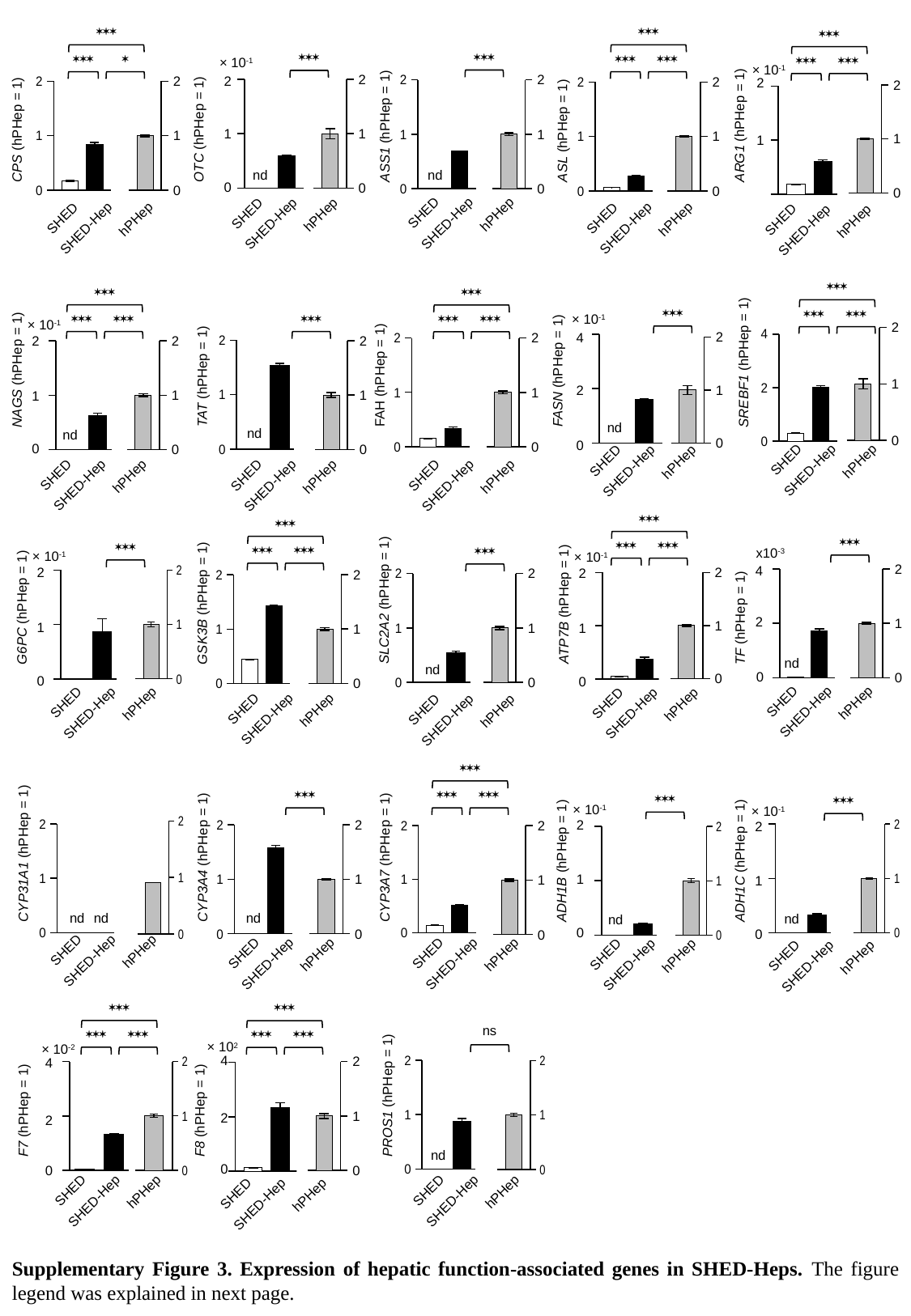

***
*
***
***
***
***
***
***
***
***
***
× 10-1
× 10-1
2
2
### Chart
| Category | | |
|---|---|---|
### Chart
| Category | /18s |
|---|---|
| HepG2 | 1.0 |
### Chart
| Category | | |
|---|---|---|
### Chart
| Category | /18s |
|---|---|
| HepG2 | 1.0 |
### Chart
| Category | | |
|---|---|---|
### Chart
| Category | /18s |
|---|---|
| HepG2 | 1.0 |
### Chart
| Category | | |
|---|---|---|
### Chart
| Category | /18s |
|---|---|
| HepG2 | 1.0 |
### Chart
| Category | /18s |
|---|---|
| HepG2 | 1.0 |
### Chart
| Category | | |
|---|---|---|ARG1 (hPHep = 1)
ASS1 (hPHep = 1)
OTC (hPHep = 1)
CPS (hPHep = 1)
ASL (hPHep = 1)
1
1
nd
nd
0
SHED
hPHep
SHED-Hep
SHED
hPHep
SHED-Hep
SHED
hPHep
SHED-Hep
SHED
hPHep
SHED-Hep
SHED
hPHep
SHED-Hep
***
***
***
***
***
***
***
***
***
***
***
× 10-1
× 10-1
### Chart
| Category | /18s |
|---|---|
| HepG2 | 1.0 |4
### Chart
| Category | | |
|---|---|---|
### Chart
| Category | | |
|---|---|---|2
### Chart
| Category | /18s |
|---|---|
| HepG2 | 1.0 |
### Chart
| Category | | |
|---|---|---|
### Chart
| Category | /18s |
|---|---|
| HepG2 | 1.0 |
### Chart
| Category | | |
|---|---|---|
### Chart
| Category | /18s |
|---|---|
| HepG2 | 1.0 |
### Chart
| Category | /18s |
|---|---|
| HepG2 | 1.0 |
### Chart
| Category | | |
|---|---|---|SREBF1 (hPHep = 1)
NAGS (hPHep = 1)
FASN (hPHep = 1)
FAH (hPHep = 1)
TAT (hPHep = 1)
2
1
nd
nd
nd
0
0
SHED
hPHep
SHED-Hep
SHED
hPHep
SHED-Hep
SHED
hPHep
SHED-Hep
SHED
hPHep
SHED-Hep
SHED
hPHep
SHED-Hep
***
***
***
***
***
***
***
***
***
x10-3
× 10-1
× 10-1
4
2
2
### Chart
| Category | | |
|---|---|---|
### Chart
| Category | /18s |
|---|---|
| HepG2 | 1.0 |
### Chart
| Category | | |
|---|---|---|
### Chart
| Category | |
|---|---|
### Chart
| Category | | |
|---|---|---|
### Chart
| Category | /18s |
|---|---|
| HepG2 | 1.0 |
### Chart
| Category | | |
|---|---|---|
### Chart
| Category | /18s |
|---|---|
| HepG2 | 1.0 |
### Chart
| Category | | |
|---|---|---|
### Chart
| Category | /18s |
|---|---|
| HepG2 | 1.0 |SLC2A2 (hPHep = 1)
GSK3B (hPHep = 1)
ATP7B (hPHep = 1)
G6PC (hPHep = 1)
TF (hPHep = 1)
2
1
1
nd
nd
0
0
0
SHED
hPHep
SHED-Hep
SHED
hPHep
SHED-Hep
SHED
hPHep
SHED-Hep
SHED
hPHep
SHED-Hep
SHED
hPHep
SHED-Hep
***
***
***
***
***
***
× 10-1
× 10-1
2
2
### Chart
| Category | |
|---|---|
### Chart
| Category | | |
|---|---|---|
### Chart
| Category | | |
|---|---|---|
### Chart
| Category | |
|---|---|
### Chart
| Category | | |
|---|---|---|
### Chart
| Category | /18s |
|---|---|
| HepG2 | 1.0 |
### Chart
| Category | | |
|---|---|---|
### Chart
| Category | /18s |
|---|---|
| HepG2 | 1.0 |
### Chart
| Category | | |
|---|---|---|
### Chart
| Category | |
|---|---|CYP31A1 (hPHep = 1)
CYP3A4 (hPHep = 1)
CYP3A7 (hPHep = 1)
ADH1C (hPHep = 1)
ADH1B (hPHep = 1)
1
1
nd
nd
nd
nd
nd
0
0
SHED
hPHep
SHED-Hep
SHED
hPHep
SHED-Hep
SHED
hPHep
SHED-Hep
SHED
hPHep
SHED-Hep
SHED
hPHep
SHED-Hep
***
***
***
× 10-2
4
### Chart
| Category | | |
|---|---|---|
### Chart
| Category | |
|---|---|2
0
SHED
hPHep
SHED-Hep
***
***
***
ns
× 102
4
### Chart
| Category | | |
|---|---|---|
### Chart
| Category | |
|---|---|
### Chart
| Category | /18s |
|---|---|
| HepG2 | 1.0 |
### Chart
| Category | | |
|---|---|---|PROS1 (hPHep = 1)
F7 (hPHep = 1)
F8 (hPHep = 1)
2
nd
0
SHED
hPHep
SHED-Hep
SHED
hPHep
SHED-Hep
Supplementary Figure 3. Expression of hepatic function-associated genes in SHED-Heps. The figure legend was explained in next page.

## Slide 4
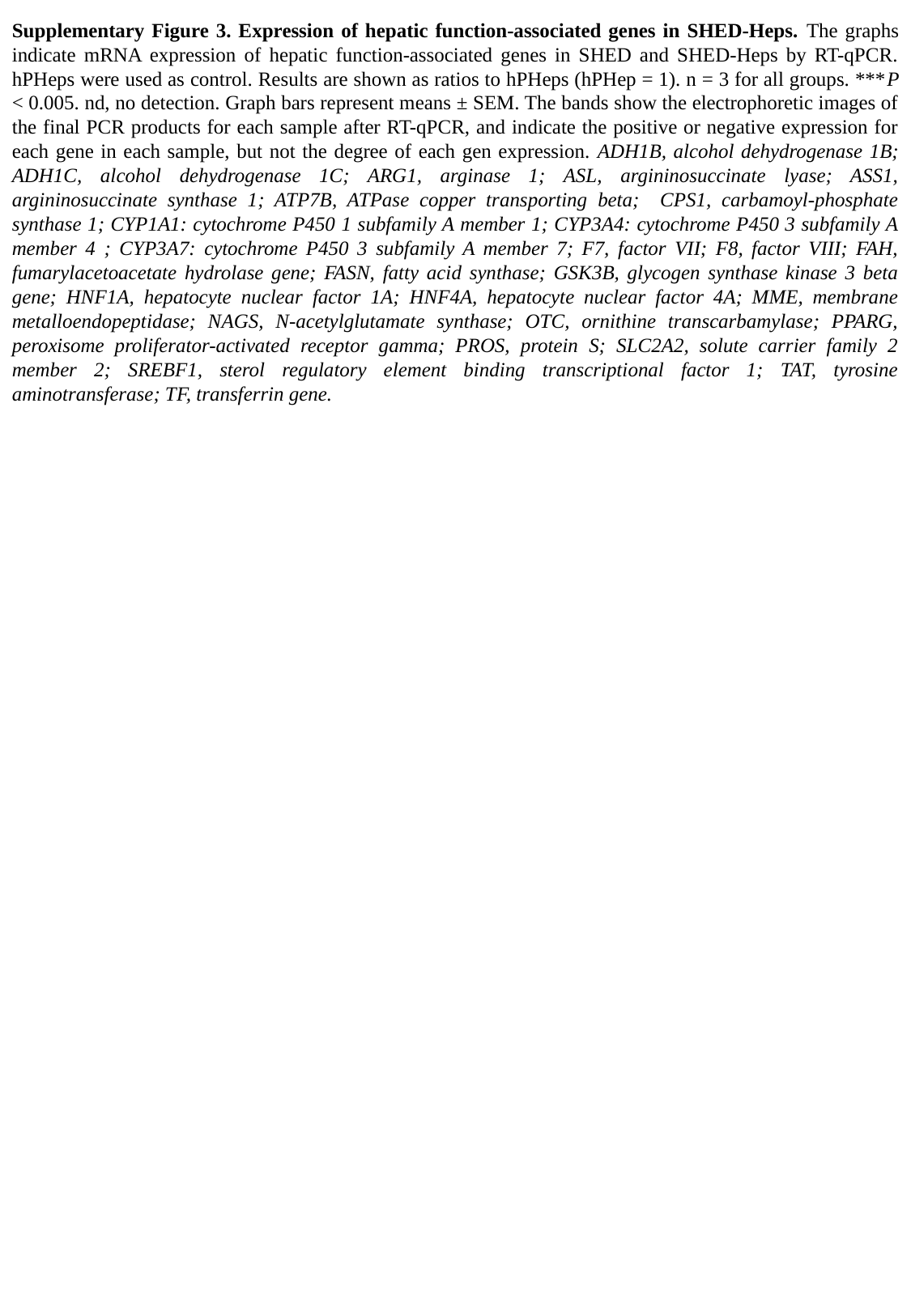

Supplementary Figure 3. Expression of hepatic function-associated genes in SHED-Heps. The graphs indicate mRNA expression of hepatic function-associated genes in SHED and SHED-Heps by RT-qPCR. hPHeps were used as control. Results are shown as ratios to hPHeps (hPHep = 1). n = 3 for all groups. ***P < 0.005. nd, no detection. Graph bars represent means ± SEM. The bands show the electrophoretic images of the final PCR products for each sample after RT-qPCR, and indicate the positive or negative expression for each gene in each sample, but not the degree of each gen expression. ADH1B, alcohol dehydrogenase 1B; ADH1C, alcohol dehydrogenase 1C; ARG1, arginase 1; ASL, argininosuccinate lyase; ASS1, argininosuccinate synthase 1; ATP7B, ATPase copper transporting beta; CPS1, carbamoyl-phosphate synthase 1; CYP1A1: cytochrome P450 1 subfamily A member 1; CYP3A4: cytochrome P450 3 subfamily A member 4 ; CYP3A7: cytochrome P450 3 subfamily A member 7; F7, factor VII; F8, factor VIII; FAH, fumarylacetoacetate hydrolase gene; FASN, fatty acid synthase; GSK3B, glycogen synthase kinase 3 beta gene; HNF1A, hepatocyte nuclear factor 1A; HNF4A, hepatocyte nuclear factor 4A; MME, membrane metalloendopeptidase; NAGS, N-acetylglutamate synthase; OTC, ornithine transcarbamylase; PPARG, peroxisome proliferator-activated receptor gamma; PROS, protein S; SLC2A2, solute carrier family 2 member 2; SREBF1, sterol regulatory element binding transcriptional factor 1; TAT, tyrosine aminotransferase; TF, transferrin gene.

## Slide 5
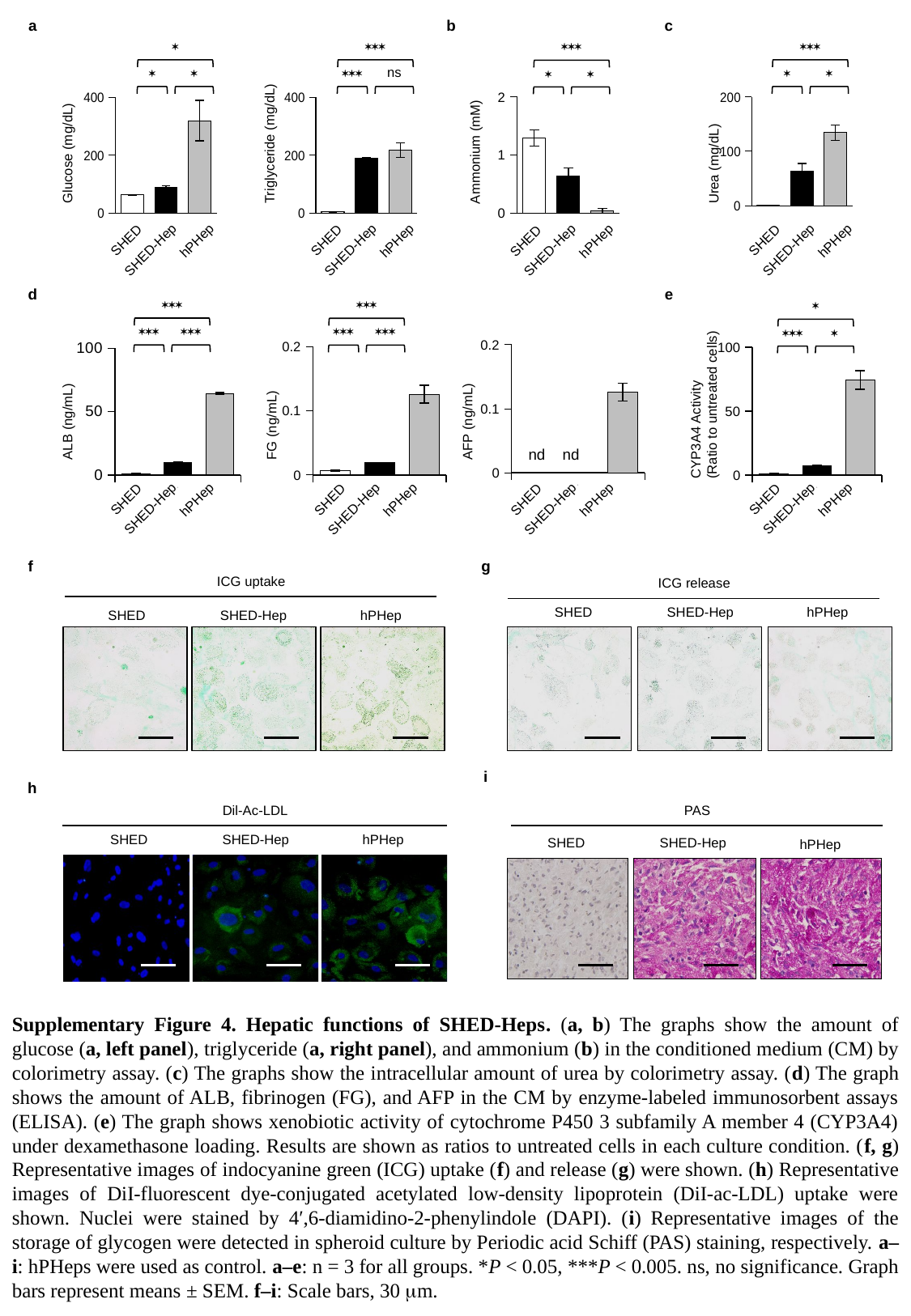

c
b
a
*
*
*
### Chart
| Category | | | |
|---|---|---|---|Glucose (mg/dL)
SHED
hPHep
SHED-Hep
***
ns
***
### Chart
| Category | | | |
|---|---|---|---|Triglyceride (mg/dL)
SHED
hPHep
SHED-Hep
***
*
*
Urea (mg/dL)
SHED
hPHep
SHED-Hep
***
*
*
### Chart
| Category | | | |
|---|---|---|---|Ammonium (mM)
SHED
hPHep
SHED-Hep
### Chart
| Category | | | |
|---|---|---|---|d
e
***
***
***
***
***
***
*
*
***
### Chart
| Category | | | |
|---|---|---|---|
### Chart
| Category | | | |
|---|---|---|---|
### Chart
| Category | | | |
|---|---|---|---|
### Chart
| Category | | | |
|---|---|---|---|CYP3A4 Activity
(Ratio to untreated cells)
ALB (ng/mL)
AFP (ng/mL)
FG (ng/mL)
nd
nd
SHED
hPHep
SHED-Hep
SHED
hPHep
SHED-Hep
SHED
hPHep
SHED-Hep
SHED
hPHep
SHED-Hep
f
g
ICG uptake
ICG release
SHED
SHED-Hep
hPHep
SHED
SHED-Hep
hPHep
i
h
PAS
SHED
SHED-Hep
hPHep
Dil-Ac-LDL
SHED
SHED-Hep
hPHep
Supplementary Figure 4. Hepatic functions of SHED-Heps. (a, b) The graphs show the amount of glucose (a, left panel), triglyceride (a, right panel), and ammonium (b) in the conditioned medium (CM) by colorimetry assay. (c) The graphs show the intracellular amount of urea by colorimetry assay. (d) The graph shows the amount of ALB, fibrinogen (FG), and AFP in the CM by enzyme-labeled immunosorbent assays (ELISA). (e) The graph shows xenobiotic activity of cytochrome P450 3 subfamily A member 4 (CYP3A4) under dexamethasone loading. Results are shown as ratios to untreated cells in each culture condition. (f, g) Representative images of indocyanine green (ICG) uptake (f) and release (g) were shown. (h) Representative images of DiI-fluorescent dye-conjugated acetylated low-density lipoprotein (DiI-ac-LDL) uptake were shown. Nuclei were stained by 4′,6-diamidino-2-phenylindole (DAPI). (i) Representative images of the storage of glycogen were detected in spheroid culture by Periodic acid Schiff (PAS) staining, respectively. a–i: hPHeps were used as control. a–e: n = 3 for all groups. *P < 0.05, ***P < 0.005. ns, no significance. Graph bars represent means ± SEM. f–i: Scale bars, 30 mm.

## Slide 6
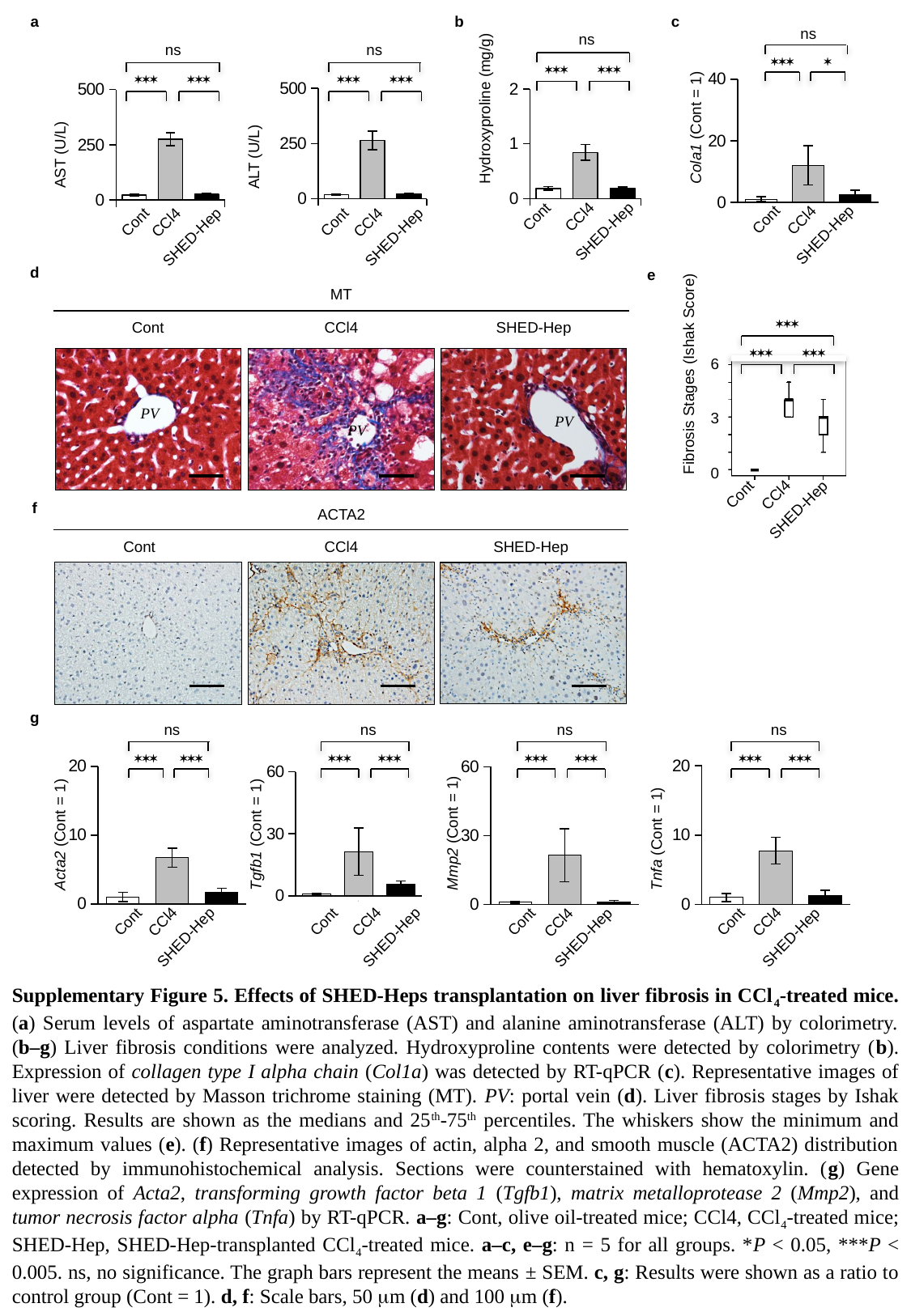

a
b
c
ns


ns

**
ns

**
ns

**
### Chart
| Category | | | |
|---|---|---|---|
### Chart
| Category | | | |
|---|---|---|---|
### Chart
| Category | | | |
|---|---|---|---|
### Chart
| Category | | | |
|---|---|---|---|Hydroxyproline (mg/g)
Cola1 (Cont = 1)
AST (U/L)
ALT (U/L)
Cont
CCl4
Cont
CCl4
Cont
Cont
CCl4
CCl4
SHED-Hep
SHED-Hep
SHED-Hep
SHED-Hep
d
e
MT
Cont
CCl4
SHED-Hep
PV
PV
PV



6
3
0
Fibrosis Stages (Ishak Score)
Cont
CCl4
f
ACTA2
SHED-Hep
Cont
CCl4
SHED-Hep
g
ns

**
ns

**
ns

**
ns

**
### Chart
| Category | | | |
|---|---|---|---|
### Chart
| Category | | | |
|---|---|---|---|
### Chart
| Category | | | |
|---|---|---|---|
### Chart
| Category | | | |
|---|---|---|---|Mmp2 (Cont = 1)
Tnfa (Cont = 1)
Acta2 (Cont = 1)
Tgfb1 (Cont = 1)
Cont
Cont
Cont
CCl4
Cont
CCl4
CCl4
CCl4
SHED-Hep
SHED-Hep
SHED-Hep
SHED-Hep
Supplementary Figure 5. Effects of SHED-Heps transplantation on liver fibrosis in CCl4-treated mice. (a) Serum levels of aspartate aminotransferase (AST) and alanine aminotransferase (ALT) by colorimetry. (b–g) Liver fibrosis conditions were analyzed. Hydroxyproline contents were detected by colorimetry (b). Expression of collagen type I alpha chain (Col1a) was detected by RT-qPCR (c). Representative images of liver were detected by Masson trichrome staining (MT). PV: portal vein (d). Liver fibrosis stages by Ishak scoring. Results are shown as the medians and 25th-75th percentiles. The whiskers show the minimum and maximum values (e). (f) Representative images of actin, alpha 2, and smooth muscle (ACTA2) distribution detected by immunohistochemical analysis. Sections were counterstained with hematoxylin. (g) Gene expression of Acta2, transforming growth factor beta 1 (Tgfb1), matrix metalloprotease 2 (Mmp2), and tumor necrosis factor alpha (Tnfa) by RT-qPCR. a–g: Cont, olive oil-treated mice; CCl4, CCl4-treated mice; SHED-Hep, SHED-Hep-transplanted CCl4-treated mice. a–c, e–g: n = 5 for all groups. *P < 0.05, ***P < 0.005. ns, no significance. The graph bars represent the means ± SEM. c, g: Results were shown as a ratio to control group (Cont = 1). d, f: Scale bars, 50 mm (d) and 100 mm (f).

## Slide 7
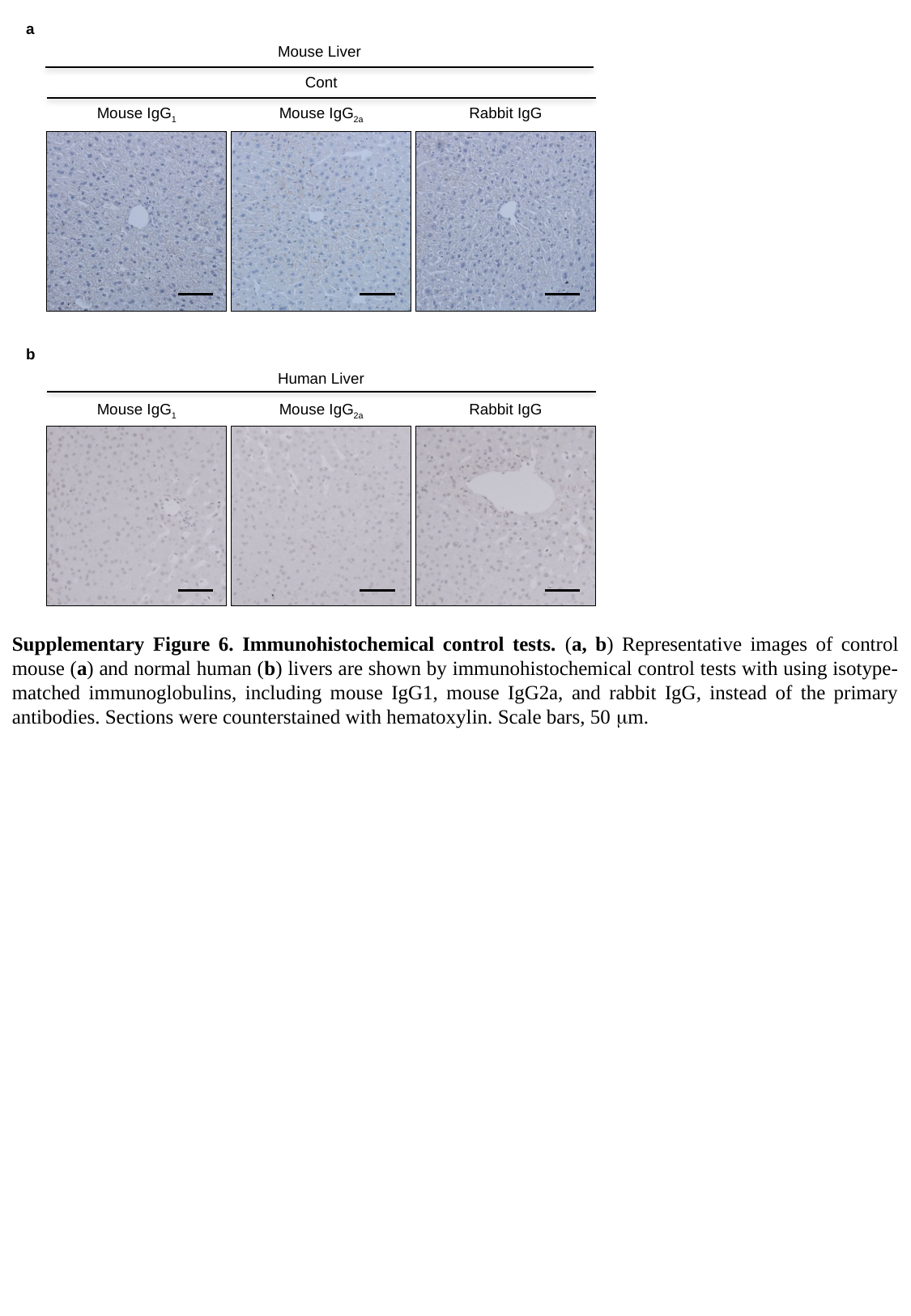

a
Mouse Liver
Cont
Rabbit IgG
Mouse IgG1
Mouse IgG2a
b
Human Liver
Rabbit IgG
Mouse IgG1
Mouse IgG2a
Supplementary Figure 6. Immunohistochemical control tests. (a, b) Representative images of control mouse (a) and normal human (b) livers are shown by immunohistochemical control tests with using isotype-matched immunoglobulins, including mouse IgG1, mouse IgG2a, and rabbit IgG, instead of the primary antibodies. Sections were counterstained with hematoxylin. Scale bars, 50 mm.

## Slide 8
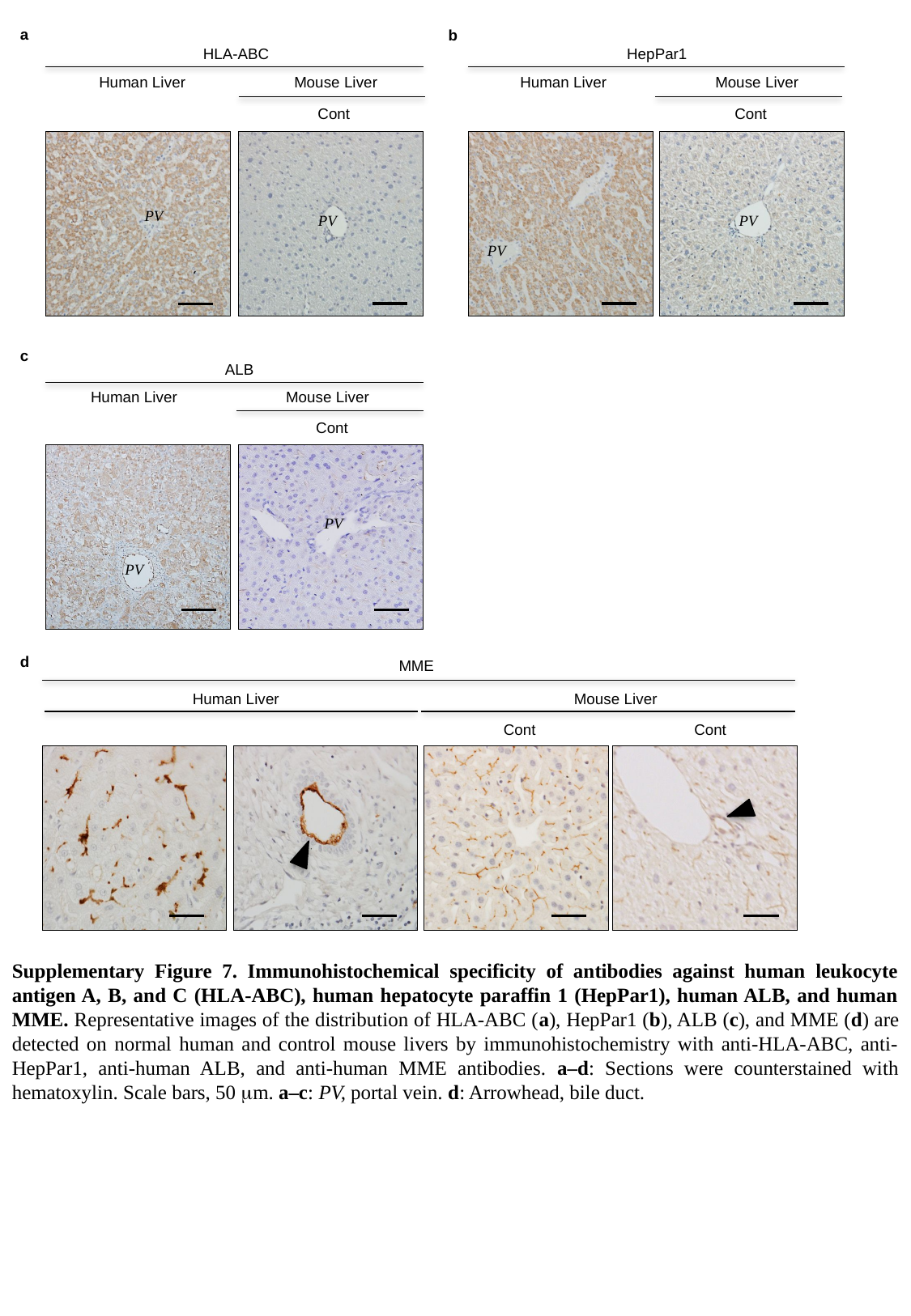

a
b
HLA-ABC
HepPar1
Human Liver
Mouse Liver
Human Liver
Mouse Liver
Cont
Cont
PV
PV
PV
PV
c
ALB
Human Liver
Mouse Liver
Cont
PV
PV
d
MME
Human Liver
Mouse Liver
Cont
Cont
Supplementary Figure 7. Immunohistochemical specificity of antibodies against human leukocyte antigen A, B, and C (HLA-ABC), human hepatocyte paraffin 1 (HepPar1), human ALB, and human MME. Representative images of the distribution of HLA-ABC (a), HepPar1 (b), ALB (c), and MME (d) are detected on normal human and control mouse livers by immunohistochemistry with anti-HLA-ABC, anti-HepPar1, anti-human ALB, and anti-human MME antibodies. a–d: Sections were counterstained with hematoxylin. Scale bars, 50 mm. a–c: PV, portal vein. d: Arrowhead, bile duct.

## Slide 9
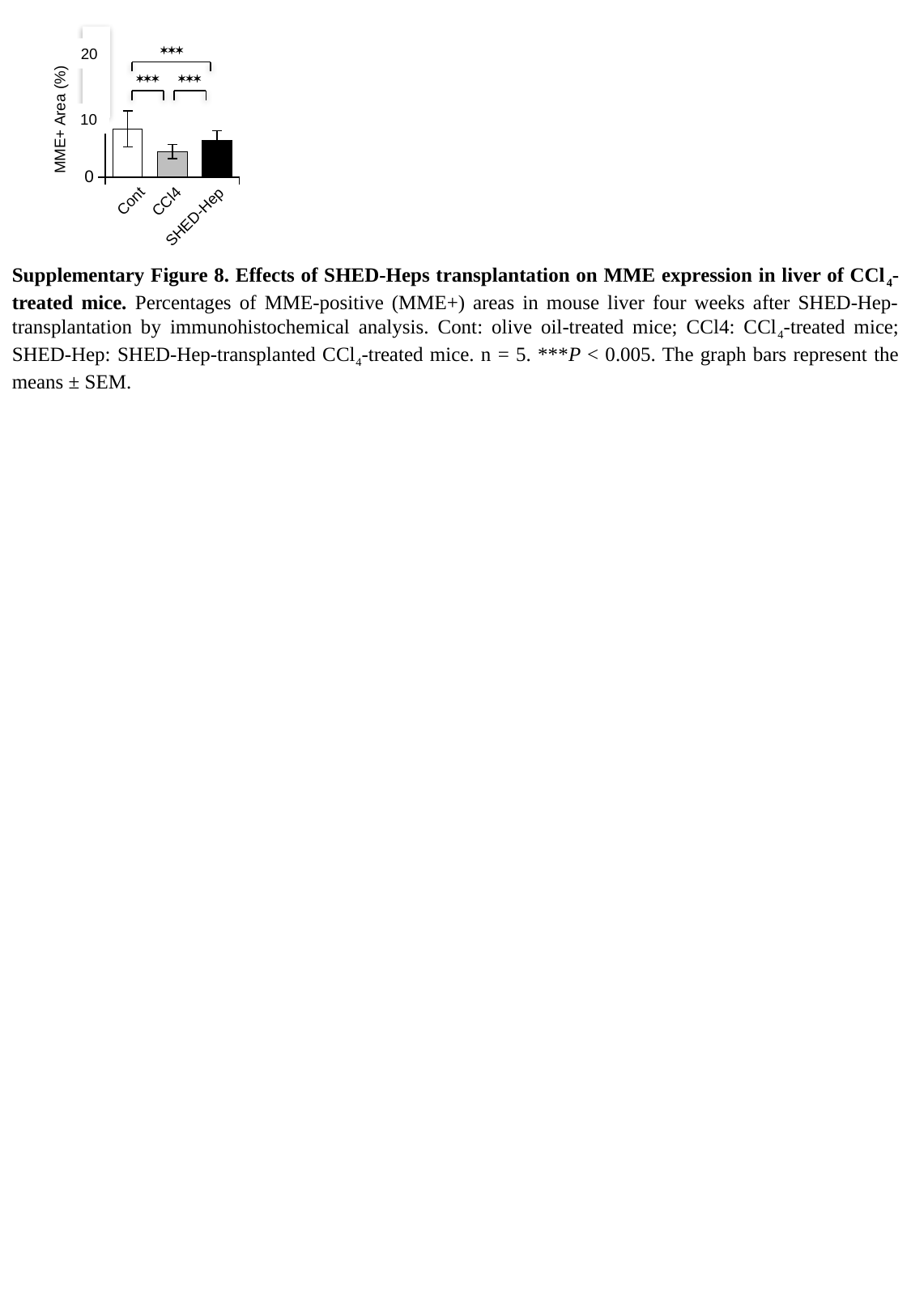




20
### Chart
| Category | | | |
|---|---|---|---|MME+ Area (%)
10
Cont
CCl4
SHED-Hep
Supplementary Figure 8. Effects of SHED-Heps transplantation on MME expression in liver of CCl4-treated mice. Percentages of MME-positive (MME+) areas in mouse liver four weeks after SHED-Hep-transplantation by immunohistochemical analysis. Cont: olive oil-treated mice; CCl4: CCl4-treated mice; SHED-Hep: SHED-Hep-transplanted CCl4-treated mice. n = 5. ***P < 0.005. The graph bars represent the means ± SEM.

## Slide 10
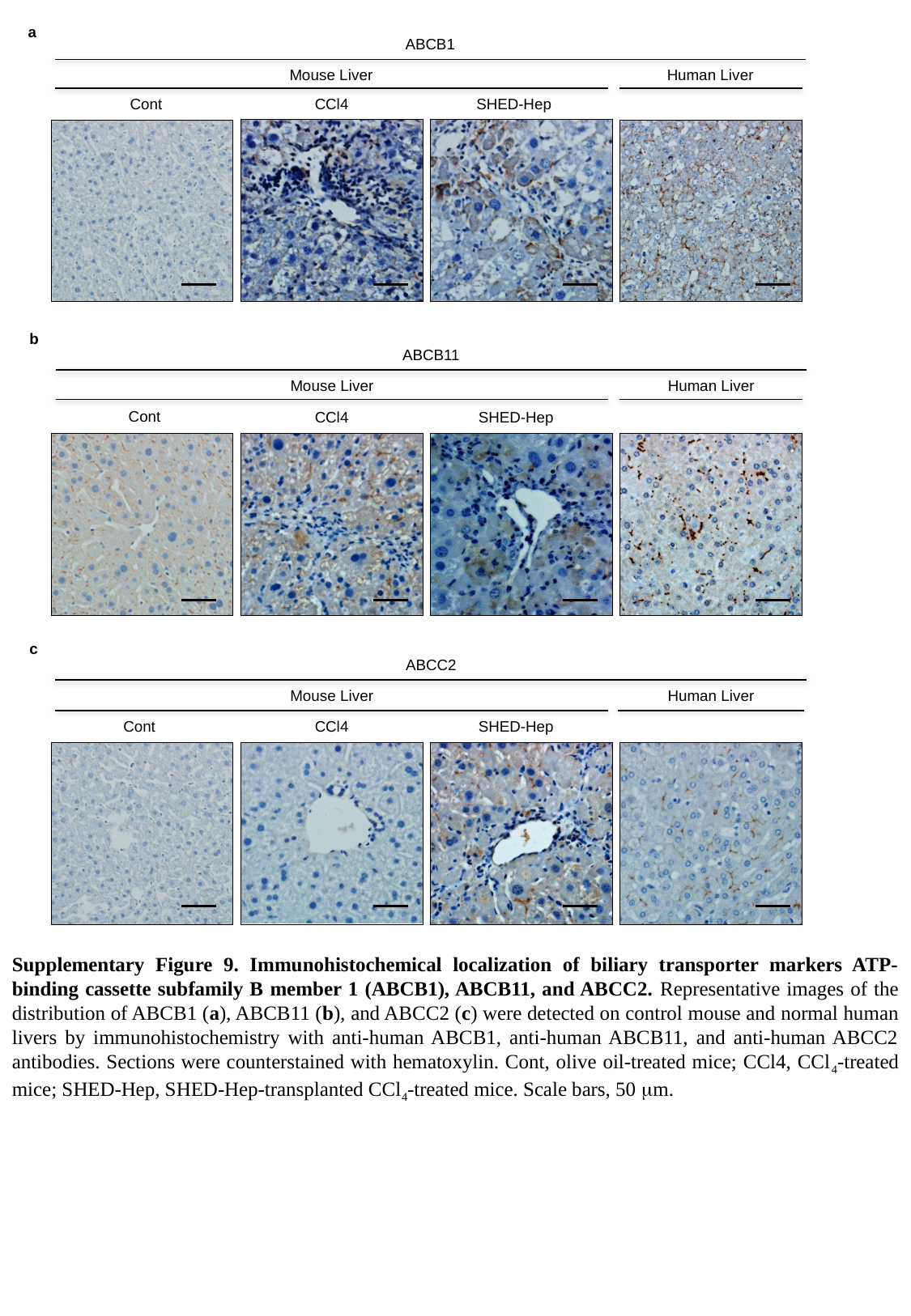

a
ABCB1
Mouse Liver
Human Liver
Cont
CCl4
SHED-Hep
b
ABCB11
Mouse Liver
Human Liver
Cont
CCl4
SHED-Hep
c
ABCC2
Mouse Liver
Human Liver
Cont
CCl4
SHED-Hep
Supplementary Figure 9. Immunohistochemical localization of biliary transporter markers ATP-binding cassette subfamily B member 1 (ABCB1), ABCB11, and ABCC2. Representative images of the distribution of ABCB1 (a), ABCB11 (b), and ABCC2 (c) were detected on control mouse and normal human livers by immunohistochemistry with anti-human ABCB1, anti-human ABCB11, and anti-human ABCC2 antibodies. Sections were counterstained with hematoxylin. Cont, olive oil-treated mice; CCl4, CCl4-treated mice; SHED-Hep, SHED-Hep-transplanted CCl4-treated mice. Scale bars, 50 mm.

## Slide 11
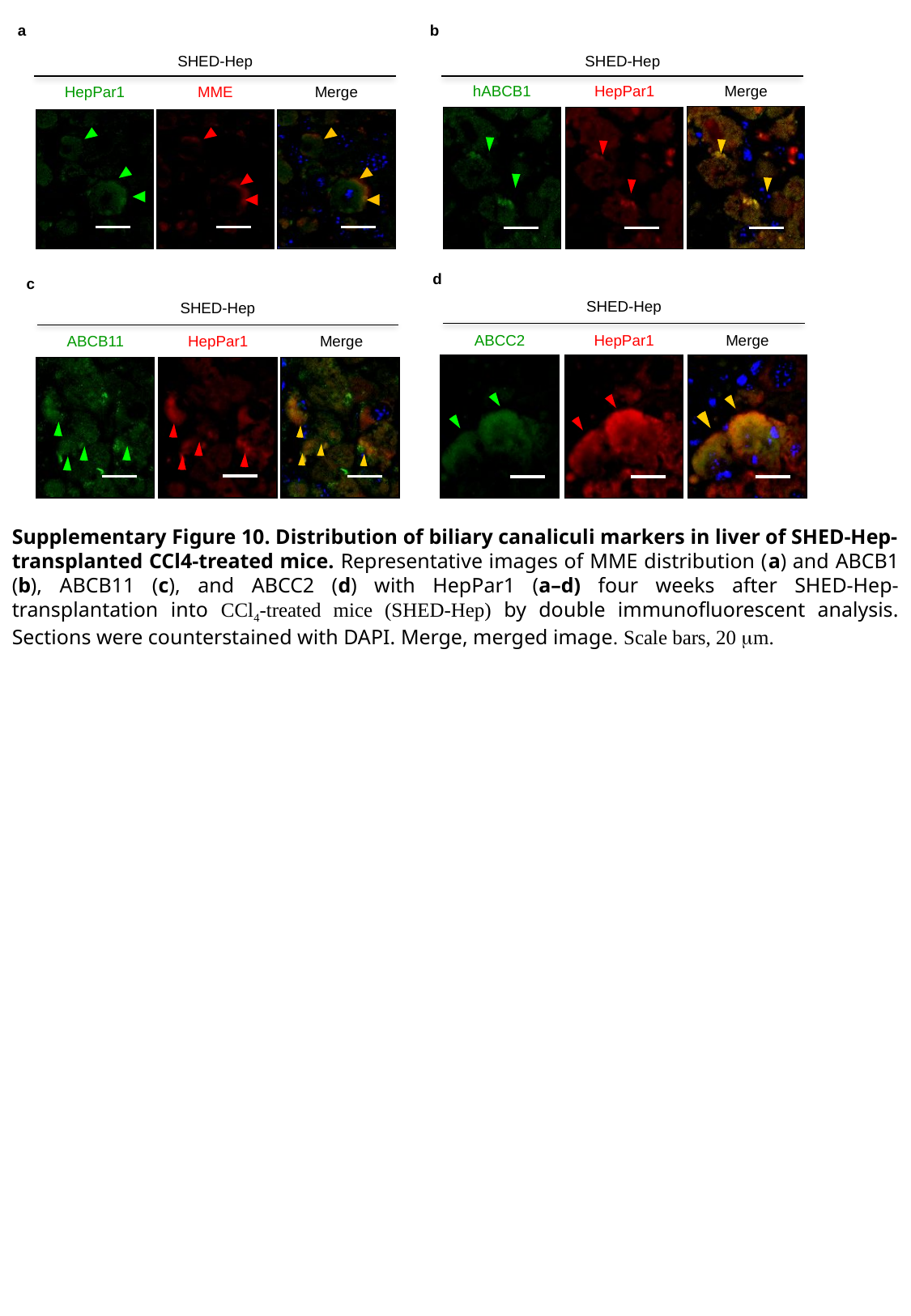

a
b
SHED-Hep
SHED-Hep
hABCB1
HepPar1
Merge
HepPar1
MME
Merge
d
c
SHED-Hep
SHED-Hep
ABCC2
HepPar1
Merge
ABCB11
HepPar1
Merge
Supplementary Figure 10. Distribution of biliary canaliculi markers in liver of SHED-Hep-transplanted CCl4-treated mice. Representative images of MME distribution (a) and ABCB1 (b), ABCB11 (c), and ABCC2 (d) with HepPar1 (a–d) four weeks after SHED-Hep- transplantation into CCl4-treated mice (SHED-Hep) by double immunofluorescent analysis. Sections were counterstained with DAPI. Merge, merged image. Scale bars, 20 mm.

## Slide 12
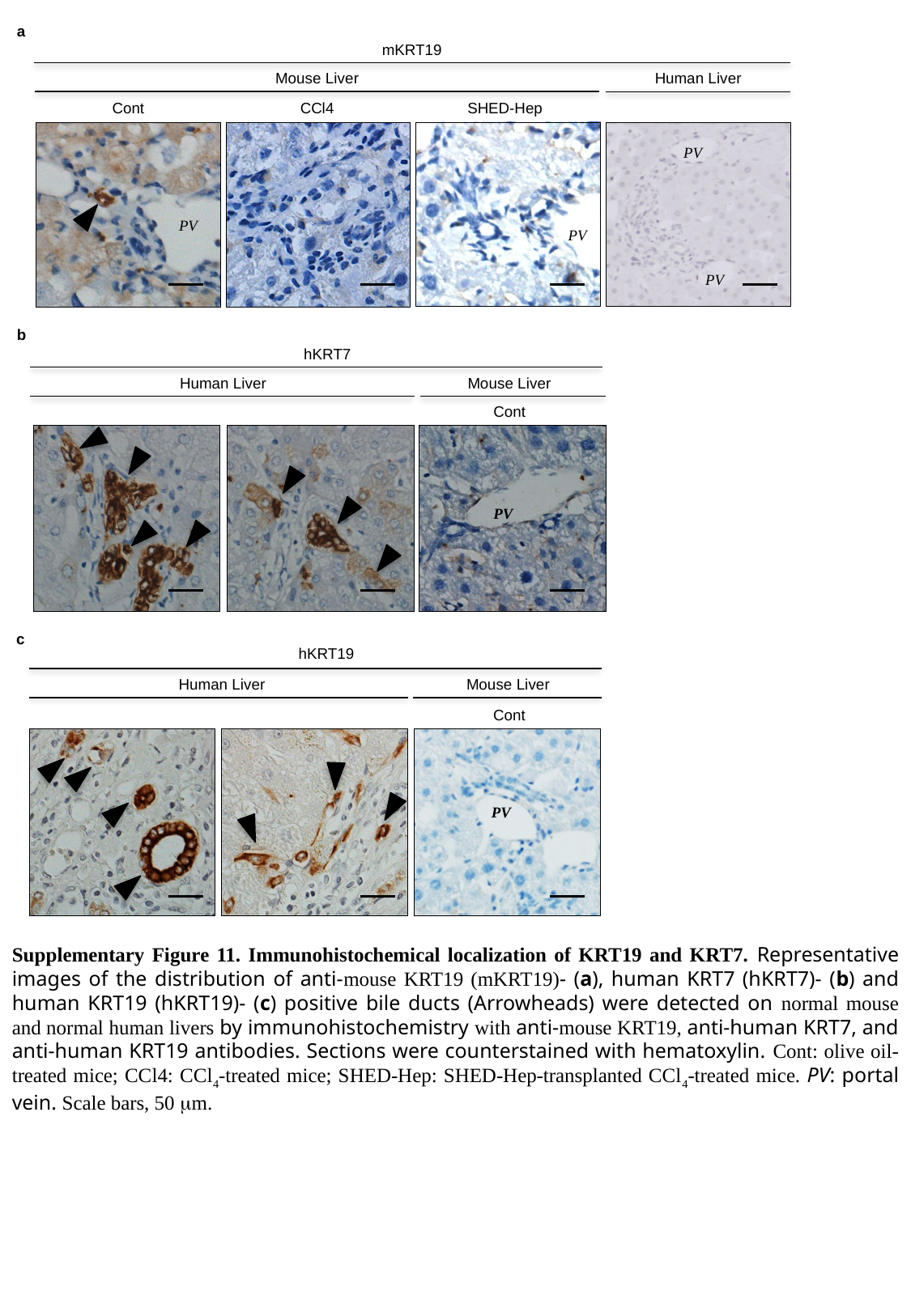

a
mKRT19
Mouse Liver
Human Liver
SHED-Hep
CCl4
Cont
PV
PV
PV
PV
b
hKRT7
Human Liver
Mouse Liver
Cont
PV
c
hKRT19
Human Liver
Mouse Liver
Cont
PV
Supplementary Figure 11. Immunohistochemical localization of KRT19 and KRT7. Representative images of the distribution of anti-mouse KRT19 (mKRT19)- (a), human KRT7 (hKRT7)- (b) and human KRT19 (hKRT19)- (c) positive bile ducts (Arrowheads) were detected on normal mouse and normal human livers by immunohistochemistry with anti-mouse KRT19, anti-human KRT7, and anti-human KRT19 antibodies. Sections were counterstained with hematoxylin. Cont: olive oil-treated mice; CCl4: CCl4-treated mice; SHED-Hep: SHED-Hep-transplanted CCl4-treated mice. PV: portal vein. Scale bars, 50 mm.
