# Supplementary material for: Cholangiogenic potential of human deciduous pulp stem cell-converted hepatocyte-like cells
Source: Stem Cell Res Ther. 2021 Jan 13;12:57. doi: 10.1186/s13287-020-02113-8 (PMC7805240; doi:10.1186/s13287-020-02113-8)
Supplement: Supplementary file 1 — Additional file 1. Supplementary Methods. Supplementary References. Supplementary Table 1. The list of specific antibodies used for flow cytometry. Supplementary Table 2. Specific antibodies for immunohistochemistry and immunofluorescence. Supplementary Table 3. List of TaqMan probes for human genes. Supplementary Table 4. List of TaqMan probes for mouse genes. Supplementary Fig. 1. Characterization of stem cells from human exfoliated deciduous teeth (SHED). Supplementary Fig. 2. Hepatogenic properties of SHED. Supplementary Fig. 3. Expression of hepatic function-associated genes in SHED-Heps. Supplementary Fig. 4. Hepatic functions of SHED-Heps. Supplementary Fig. 5. Effects of SHED-Heps transplantation on liver fibrosis in CCl4-treated mice. Supplementary Fig. 6. Immunohistochemical control tests. Supplementary Fig. 7. Immunohistochemical specificity of antibodies against human leukocyte antigen A, B, and C (HLA-ABC), human hepatocyte paraffin 1 (HepPar1), human ALB, and human MME. Supplementary Fig. 8. Effects of SHED-Heps transplantation on MME expression in liver of CCl4-treated mice. Supplementary Fig. 9. Immunohistochemical localization of biliary transporter markers ATP-binding cassette subfamily B member 1 (ABCB1), ABCB11, and ABCC2. Supplementary Fig. 10. Distribution of biliary canaliculi markers in liver of SHED-Hep-transplanted CCl4-treated mice. Supplementary Fig. 11. Immunohistochemical localization of KRT19 and KRT7. [file 13287_2020_2113_MOESM1_ESM.zip › Ratih(SHEDHepBiliaryNetwork)SupplInformationSCRT_Revised.docx]

**Supplementary Information**

**Cholangiogenic potential of human deciduous pulp stem cell-converted hepatocyte-like cells**

Ratih Yuniartha, Takayoshi Yamaza, Soichiro Sonoda, Koichiro Yoshimaru, Toshiharu Matsuura, Haruyoshi Yamaza, Yoshinao Oda, Shouichi Ohga, Tomoaki Taguchi

**Supplementary Methods**

*Isolation, culture, and characterization of stem cells from human exfoliated deciduous teeth (SHED)*

Dental pulp tissues of human exfoliated deciduous teeth (n = 3) were digested with 0.3% collagenase type I (Worthington Biochemicals, Lakewood, NJ, USA) and 0.4% dispase II (Sanko Junyaku, Tokyo, Japan) for 60 min at 37^o^C and passed through a 70-μm cell strainer (Corning, Corning, NY, USA). The obtained cells were seeded at 1.0 – 1.5 × 10^7^ cells on a T-75 culture flask (Corning). Three hours after seeding, the cultures were washed with sterilized phosphate-buffered saline (PBS). The adherent cells were grown in a complete medium until forming colony-forming unit fibroblasts (CFU-F) as described before [1,2]. The complete medium consisted of 15% fetal bovine serum (FBS; Equitech-Bio, Kerrville, TX, USA), 100 μM L-ascorbic acid 2-phosphate (Wako Pure Chemicals, Osaka, Japan), 2 mM L-glutamine (Nacalai Tesque, Kyoto, Japan), and premixed antibiotics containing 100 U/mL penicillin and 100 μg/mL streptomycin (Nacalai Tesque) in minimum Eagle's medium alpha modification (αMEM; Thermo Fisher Scientific, Waltham, MA). The attached colonies were formed fourteen days. The attached CFU-F-forming cells were passaged three times at 4 × 10^3^ cells/cm^2^. The passage 3 (P3) cells were used for further experiments. The medium was changed twice a week. SHED was characterized by the criteria of mesenchymal stem/stromal cells (MSCs) [3], including plastic adherence, surface antigen expression, and multilineage osteogenic, chondrogenic, and adipogenic differentiation, according to previous studies [4,5]. Cell proliferation of SHED were analyzed by bromodeoxyuridine (BrdU)-uptake and population doubling assays [5]

*Morphology of SHED-derived hepatic committed cells (SHED-Hep)*

After SHED were cultured under a hepatogenic condition, SHED-Heps were fixed with 2% paraformaldehyde and 0.1% toluidine blue solution overnight in Dulbecco’s PBS (Nacalai Tasque), and observed with a vertical light microscope AxioVert (Carl Zeiss Microscopy, Jena, Germany). As controls, intact SHED and human primary hepatocytes (hPHep; Lonza, Basel, Switzerland) were used.

*Localization of human albumin and glycogen in SHED-Heps*

Human albumin (ALB) and glycogen in SHED-Heps were analyzed in paraffin section of SHED-Hep spheroids by immunofluorescent assay and Periodic acid-Schiff staining, respectively, and observed with a upright light microscope Axio Imager M2 (Carl Zeiss Microscopy). As controls, intact SHED and hPHep (Lonza) were used.

*Target gene expression analysis of SHED-Hep*

Target gene expression analysis in SHED-Heps were performed by reverse transcription-quantitative polymerase chain reaction (RT-qPCR). Total RNA was extracted from the cultured SHED-Heps and analyzed for RT-qPCR, as described below. As controls, intact SHED and hPHep (Lonza) were used. All the tests were carried out, at least, in triplicate.

*Glucose, triglyceride, ALB, fibrinogen, and alpha fetoprotein secretion assays*

Cultured SHED-Heps were incubated for 48 h in a freshly-prepared culture medium containing FGF2 (10 ng/mL; PeproTech, Rocky Hill, NJ), HGF (20 ng/mL; PeproTech), and nicotinamide (0.61 g/L; Merck, Darmstadt, Germany) in Iscove's Modified Dulbecco's Media (IMDM; Thermo Fisher Scientific). The collected conditioned medium (CM) was quickly applied to colorimetric assay and enzyme linked immunosorbent assay (ELISA) using Glucose CII-test (Wako Pure Chemicals), Triglyceride E-test (Wako Pure Chemicals), and AssayMAX Human Albumin ELISA Kit (AssayPro, St. Charles, MO), Fibrinogen Human SimpleStep ELISA Kit (Abcam, Cambridge, UK), and Alpha Fetoprotein Human SimpleStep ELISA kit (Abcam), respectively, according to the manufacturers’ instructions. As controls, intact SHED and hPHep (Lonza) were cultured as described above and each CM was used. All the tests were carried out, at least, in triplicate.

*Ammonia metabolism assay*

SHED-Hep were washed with Hanks’ balanced salt solution without phenol red (HBSS; Nacalai Tasque) and incubated with ammonium chloride (1 mM; Nacalai Tasque) in HBSS (Nacalai Tesque) for 24 h. The CM was collected and quickly used for measuring ammonia using a colorimetric ammonia assay kit (BioVision, Milpitas, CA) according to the manufacturer’s instructions.. As controls, intact SHED and hPHep (Lonza) were used. All the tests were carried out, at least, in triplicate.

*Intracellular urea content assay*

SHED-Heps were washed with HBSS without phenol red (Nacalai Tesque) and cultured for 24 h. The intracellular urea was measured by colorimetric assay using a Urea Assay Kit (Abcam, Cambridge, UK) according to the manufacturer’s instructions. As controls, intact SHED and hPHep (Lonza) were cultured as described above and used for the analysis. As controls, intact SHED and hPHep (Lonza) were used. All the tests were carried out in triplicate.

*Cytochrome P450 3A4 (CYP3A4) activity test*

SHED-Heps were incubated with or without dexamethasone (50 μM; Merck) for 24 h. CYP3A4 activity was analyzed by spectrometric assay using a P450-Glo CYP3A4 Kit with Luciferin-IPA (Promega, Madison, WN) according to the manufacturer’s instructions. As controls, intact SHED and hPHep (Lonza) were cultured as described above and used for the analysis. Results were measured with a luminometer GloMax Navigator (Promega) and shown as normalized relative light unit values to the untreated well in each culture condition according to the manufacturer’s instructions. As controls, intact SHED and hPHep (Lonza) were used. All the tests were carried out in triplicate.

*Indocyanin green (ICG) uptake and release assay*

SHED-Heps were incubated with IGC (Merck) in IMDM (Thermo Fisher Scientific) for 1 h according to the manufacturer’s instructions. The ICG uptake in SHED-Heps were acquired with a vertical light microscope Axio Vert (Carl Zeiss Microscopy). SHED-Heps were washed with HBSS without phenol red (Nacalai Tesque) and cultured. Then 6 hours later, the IGC release was acquired with a vertical light microscope Axio Vert (Carl Zeiss Microscopy). As controls, intact SHED and hPHep (Lonza) were used.

*Uptake of low-density lipoproteins (LDL) assay*

SHED-Heps were cultured overnight with IMDM (Thermo Fisher Scientific) containing 0.1% bovine serum albumin (BSA; Merck), and incubated for 5 hours with DiL-florescent dye-conjugated acetylated LDL (DiI-Ac-LDL; 10 μg/mL; Cell Applications, San Diego, CA) in IMDM (Thermo Fisher Scientific) containing 0.1% BSA. The cells were fixed with 4% paraformaldehyde and stained with DAPI. Fluorescent images of DiI-Ac-LDL were acquired with a fluorescent microscope Axio Imager M2 (Carl Zeiss Microscopy) equipped with an ApoTome 2 (Carl Zeiss Microscopy). As controls, intact SHED and hPHep (Lonza) were used.

*In vivo* biomarker assay of mice

Aspartate aminotransferase and alanine aminotransferase in serum were measured by colorimetric assay using Transaminase CII-Test Kit (Wako Pure Chemicals) according to the manufacturer’s instructions. For hydroxyproline content assay, mouse liver tissues were measured by colorimetric assay using Hydroxyproline Assay Kit (Biovision, Milpitas, CA, USA) according to the manufacturer’s instructions. The gene expression of mouse *actin alpha 2 smooth muscle*, *collagen type I alpha 1*, *matrix metalloprotease 2*, *transforming growth factor beta*, and *tumor necrosis factor alpha* in mouse liver tissues were examined by RT-qPCR. All the tests were carried out in triplicate.

*Histological, immunohistochemical and double immunofluorescent analyses*

Paraffin sections were treated by hematoxylin and eosin and Masson trichrome staining. Fibrosis stage was determined by Ishak scoring [8]. Five representative images from each experimental group were randomly selected. The regions of interest were measured using Image-J software (Software version 1.50; National Institutes of Health, Bethesda, MD) according to the previous study [9].

For immunohistochemistry, paraffin sections were incubated with 3% hydrogen peroxide in methanol for 30 min to quench the endogenous peroxidase activity and treated with 5% normal goat serum (Thermo Fisher Scientific) in PBS. Sections were then incubated with primary antibodies overnight at 4^o^C, followed by treating with Dako Envision+ system-HRP labeled polymer anti-rabbit or anti-mouse (Agilent, Santa Clara, CA). Finally, sections were visualized with 0.05% diaminobenzidine-4HCl (Dojindo Laboratories, Kumamoto, Japan) and 0.006% hydrogen peroxide for 5 min and counter-stained with hematoxylin. All sections were observed with a upright light microscope Axio Imager M2 (Carl Zeiss Microscopy). For double immunofluorescence, paraffin sections were blocked with 5% normal donkey serum (Thermo Fisher Scientific) and incubated with the first primary antibodies, followed by treating with Alexa Fluor 488-conjugated secondary antibodies (Thermo Fisher Scientific). Thereafter, the same sections were treated with the second primary antibodies, followed by treating with Alexa Fluor 568-conjugated secondary antibodies (Thermo Fisher Scientific). Finally, all immunofluorescent samples were stained with 4', 6-diamidino-2-phenylindole (DAPI; 1 μg/ml; Thermo Fisher Scientific). All sections were observed with a fluorescent microscope Axio Imager M2 (Carl Zeiss Microscopy) equipped with an ApoTome 2 (Carl Zeiss Microscopy). Primary antibodies used in the present immunohistochemical and immunofluorescent assays are summarized in **Supplementary Table 2**. Immunohistochemical and immunofluorescent controls for primary antibodies were incubated with non-immune immunoglobulins, including mouse IgG_1_, mouse IgG_2a_, and rabbit IgG, instead of the primary antibodies. To test the cross-reactivity of primary antibodies, paraffin sections of normal human and mouse liver tissue were immunohistochemically stained as above.

*ELISA and colorimetric assays*

Total protein concentration of samples was quantified using Bio‐Rad protein assay (Bio‐Rad Laboratories, Hercules, CA) to ensure equal loading of each well. Results from ELISA and colorimetric assay were measured with a microplate reader Multiskan GO (Thermo Fisher Scientific). All the tests were carried out in triplicate or more.

*Flow cytometric (FCM) analysis*

Cultured cells (0.1 × 10^6^/100 μL) were suspended in ice-cold FCM buffer. The FCM buffer consisted of 2% heat-inactivated FBS (Merck) in HBSS (Nacalai Tasque). The cell suspension was incubated with R-phycoerythrin (R-PE)-conjugated primary antibodies (1 μg per 100 μL) at 4^o^C for 45 min. As the controls, isotype-matched antibodies conjugated with R-PE were used instead of the primary antibodies. Stained cells were washed with FCM buffer and measured with a flow cytometer FACSVerse (BD Biosciences, Franklin Lake, NJ). The number (percentage) of positive cells was determined using FACSuite software (Software version V1.0.5.3841, BD Biosciences) compared control cells stained with corresponding isotype-matched antibodies in which a false-positive rate of less than 1% was accepted [3]. All the tests were carried out in triplicate or more. Primary antibodies used in FCM assay are summarized in **Supplementary Table 1**.

*Total RNA extraction and cDNA production*

RNA samples were extracted from cell and tissue samples using a TRIzol regent (Thermo Fisher Scientific) and digested with DNase I (Promega). Extracted RNA samples were purified using an RNeasy Mini Kit (Qiagen, Venlo, Netherland). To confirm no contamination of genome DNA, RNA samples were analyzed by PCR with the primer pair for human glyceraldehyde 3-phosphate dehydrogenase (GenBank accession no. [M33197](http://www.pnas.org/external-ref?link_type=GEN&access_num=M33197); sense, 5′-AGCCGCATCTTCTTTTGCGTC-3′ [nucleotide 12-32]; antisense, 5′-TCATATTTGGCAGGTTTTTCT-3′ [nucleotide 807-827]) on a thermal cycler T-100 (Bio-Rad Laboratories). Each amplified PCR product (5 μL) was electrophoresed in 2 % agarose gels (Nacalai Tasque). Gels were stained with 0.5 μg/mL ethidium bromide (Bio-Rad Laboratories) and captured the images on a gel imager Gel Doc Ez System (Bio-Rad Laboratories). The complementary DNA (cDNA) was prepared from the purified total RNA by reverse transcription reaction using a Revertra Ace qPCR kit (TOYOBO, Tokyo, Japan) according to the manufacturer’s instructions and used for RT-qPCR assays.

*RT-qPCR assay*

The gene expression was analyzed by RT-qPCR using the cDNA mixed with a EagleTaq Master Mix (Roche Applied Science, Babaria, Germany) and target TaqMan probes (Thermo Fisher Scientific) with a real-time PCR machine Light Cycler 96 system (Roche Applied Science). PCR steps were as follows: preincubation 1: 50℃ for 120 sec, preincubation 2: 95℃ for 600 sec, two step amplification: 95℃ for 15 sec, 60℃ for 60 sec (45 cycles). Human and mouse 18S riboxsomal RNAs were used for normalization. All the tests were carried out in triplicate or more. TaqMan probes used in RT-qPCR are summarized in **Supplementary Tables 3, 4**.

**Supplementary References**

1. Miura M, Gronthos S, Zhao M, Lu B, Fisher LW, Robey PG, et al. SHED: stem cells from human exfoliated deciduous teeth. Proc Natl Acad Sci USA. 2003;100:5807–58.
2. Yamaza T, Kentaro A, Chen C, Liu Y, Shi Y, Gronthos S, et al. Immunomodulatory properties of stem cells from human exfoliated deciduous teeth. Stem Cell Res Ther. 2010;1:5.
3. Dominici M, Le Blanc K, Mueller I, Slaper-Cortenbach I, Marini F, Krause D, et al. Minimal criteria for defining multipotent mesenchymal stromal cells. The International Society for Cellular Therapy position statement. Cytotherapy. 2006;8:315–7.
4. Ma L, Makino Y, Yamaza H, Akiyama K, Hoshino Y, Song G, et al. Cryopreserved Dental Pulp Tissues of Exfoliated Deciduous Teeth Is a Feasible Stem Cell Resource for Regenerative Medicine. PLoS One. 2012;7:e51777.
5. Iwanaka T, Yamaza T, Sonoda S, Yoshimaru K, Matsuura T, Yamaza H, et al. A model study for the manufacture and validation of clinical-grade deciduous dental pulp stem cells for chronic liver fibrosis treatment. Stem Cell Res Ther. 2020;11:134.
6. Fujiyoshi J, Yamaza H, Sonoda S, Yuniartha R, Ihara K, Nonaka K, et al. Therapeutic potential of hepatocyte-like-cells converted from stem cells from human exfoliated deciduous teeth in fulminant Wilson’s disease. Sci Rep. 2019;9:1535.
7. Saeed AI, Sharov V, White J, Li J, Liang W, Bhagabati N, Braisted J, Klapa M, Currier T, Thiagarajan M, Sturn A, Snuffin M, Rezantsev A, Popov D, Ryltsov A, Kostukovich E, Borisovsky I, Liu Z, Vinsavich A, Trush V, Quackenbush J. TM4: a free, open-source system for microarray data management and analysis. [Biotechniques. 2003;34:374–8.](http://www.tm4.org/documentation/TM4_Biotechniques_2003.pdf)
8. Ishak K, Baptista A, Bianchi L, Callea F, De Groote J, Gudat F, et al. Histological grading and staging of chronic hepatitis. J Hepatol. 1995;22:696–99.
9. Yamaza T, Miura Y, Bi Y, Liu Y, Akiyama K, Sonoyama W, et al. Pharmacologic stem cell based intervention as a new approach to osteoporosis treatment in rodents. PLoS ONE. 2008;3:e2615.

**Supplementary Table 1.** The list of specific antibodies used for flow cytometry

| **Antibody names, antigen** | **Antibody types, host, clone name** | **Supplier names** |
| --- | --- | --- |
| anti-ABCB1 antibody, human | purified IgG, rabbit | Atlas Antibodies |
| anti-ABCB11 antibody, human | purified IgG2a, mouse, M2 III-6 | Atlas Antibodies |
| anti-ANPEP antibody, human | R-PE -conjugated IgG1 kappa, mouse, IV M44 | BioLegend |
| anti-CD11b antibody, human | R-PE -conjugated IgG1 kappa, mouse, HI111 | BioLegend |
| anti-CD14 antibody, human | R-PE -conjugated IgG1 kappa, mouse, 63D3 | BioLegend |
| anti-CD19 antibody, human | R-PE -conjugated IgG1 kappa, mouse, 47G | BioLegend |
| anti-CD29 antibody, human | R-PE -conjugated IgG1 kappa, mouse, V A-S202 | BioLegend |
| anti-CD34 antibody, human | R-PE-conjugated IgG2a kappa, mouse, 561 | BioLegend |
| anti-CD45 antibody, human | R-PE-conjugated IgG1 kappa, mouse, 2D1 | BioLegend |
| anti-CD90 antibody, human | R-PE-conjugated IgG1 kappa, mouse, 5E10 | BioLegend |
| anti-CD73 antibody, human | R-PE-conjugated IgG1 kappa, mouse, AD2 | BioLegend |
| anti-CD105 antibody, human | R-PE-conjugated IgG1 kappa, mouse, 43A3 | BioLegend |
| anti-CD146 antibody, human | R-PE-conjugated IgG1 kappa, mouse, P1H112 | BioLegend |
| anti-EPCAM antibody, human | R-PE-conjugated IgG2a kappa, mouse, CO17-1A | BioLegend |
| anti-HLA-DR antibody, human | R-PE-conjugated IgG2a kappa, mouse, L243 | BioLegend |
| anti-MME antibody, human | R-PE-conjugated IgG1 kappa, mouse, V CD10.7 | BioLegend |
| anti-NCAM1 antibody, human | R-PE-conjugated IgG1 kappa, mouse, 5.1H11 | BioLegend |
| anti-PROM1 antibody, human | R-PE-conjugated IgG1 kappa, mouse, clone 7 | BioLegend |
| anti-STRO1 antibody, human | R-PE-conjugated IgM, mouse, STRO1 | Abcam |
| control mouse IgG1 kappa | R-PE-conjugated IgG1 kappa, mouse, MOPC-21 | BioLegend |
| control mouse IgG2a kappa | R-PE-conjugated IgG2a kappa, mouse, MOPC-173 | BioLegend |
| control mouse IgM | R-PE-conjugated IgM, mouse, MM-30 | BioLegend |
| control rabbit IgG | R-PE-conjugated IgG, rabbit | BioLegend |

ABCB1, ATP-binding cassette subfamily B member 1; ABCB11, ATP-binding cassette subfamily B member 11; ANPEP, alanyl aminopeptidase, membrane; EPCAM, epithelial cell adhesion molecule; HLA-DR: human leukocyte antigen DR; MME, membrane metalloendopeptidase; NCAM1, neural cell adhesion molecule; PROM1, promin 1; R-PE: R-phycoerythrin

**Supplementary Table 2.** Specific antibodies for immunohistochemistry and immunofluorescence.

| **Antibody names, antigen** | **Antibody types, host, clone name** | **Supplier names** |
| --- | --- | --- |
| anti-ABCB1 antibody, human | purified IgG, rabbit | Atlas Antibodies |
| anti-ABCB11 antibody, human | purified IgG2a, mouse, M2 III-6 | Atlas Antibodies |
| anti- ABCC2 antibody, human | purified IgG, rabbit | Abcam |
| anti-ACTA2, mouse | purified IgG2a, mouse, ASM-1/1A4 | Merck |
| anti-albumin antibody, human | purified IgG, rabbit | Abcam |
| anti-albumin antibody, mouse | purified IgG, rabbit | Abcam |
| anti-HepPar1 antibody, human | purified IgG1, mouse, OCH1E5 | Abcam |
| anti-HLA-ABC antibody, human | purified IgG2a, mouse, W6/32 | Abcam |
| anti-KRT7 antibody, human | purified IgG1, mouse, OV-TL 12/30 | Abcam |
| anti-KRT19 antibody, human | purified IgG1, mouse, RCK108 | Abcam |
| anti-KRT19 antibody, mouse | purified IgG, rabbit, EPNCIR127B | Abcam |
| anti-MME antibody, human | purified IgG1, mouse, 56C6 | Abcam |
| control mouse IgG1 kappa | purified IgG1 kappa, mouse, MOPC-21 | Abcam |
| control mouse IgG2a kappa | purified IgG2a kappa, mouse, MOPC-173 | Abcam |
| control rabbit IgG | purified IgG, rabbit | Abcam |

ABCB1, ATP-binding cassette subfamily B member 1; ABCB11, ATP-binding cassette subfamily B member 11; ABCC2, ATP-binding cassette subfamily C member 2; ACTA2, actin, alpha 2, smooth muscle; HepPar1, human hepatocyte paraffin 1; HLA-ABC, human leukocyte antigens A, B, and C; KRT7, cytokeratin 7; KRT19, cytokeratin 19; MME, membrane metalloendopeptidase

**Supplementary Table S3.** List of TaqMan probes for human genes.

| **Gene names** | **Gene assay ID Numbers** |  | **Gene names** | **Gene assay ID Numbers** |
| --- | --- | --- | --- | --- |
| *ACAN*  *ADH1B*  *ADH1C*  *AFP* | Hs00153936_m1  Hs00605175_m1  Hs00817827_m1  Hs00173490_m1 |  | *HNF1A*  *HNF4A*  *HNF6*  *KRT18* | Hs00167041_m1  Hs00230853_m1  Hs00413554_m1  Hs02827483_g1 |
| *ALB*  *ARG1*  *ASL*  *ASS1* | Hs00910225_m1  Hs00968979_m1  Hs00902699_m1  Hs01597989_g1 |  | *KRT19*  *KRT7*  *LPL*  *MME* | Hs00761767_s1  Hs00559840_m1  Hs00173425_m1  Hs00153510_m1 |
| *ATP7B*  *ABCB1*  *ABCB11*  *ABCC2*  *BGLAP* | Hs00163739_m1  Hs00184500_m1  Hs00184824_m1  Hs00166123_m1  Hs01587814_g1 |  | *NAGS*  *NR1I2*  *OTC*  *PPARA*  *PPARG* | Hs00400246_m1  Hs00243666_m1  Hs00166892_m1  Hs00231882_m1  Hs0115513_m1 |
| *CPS1* | Hs00157048_m1 |  | *PROS1* | Hs0165590_m1 |
| *CYP1A1* | Hs00153120_m1 |  | *RUNX2* | Hs00231692_m1 |
| *CYP3A4*  *CYP3A7*  *F7*  *F8* | Hs00604506_m1  Hs00426361_m1  Hs001551992_m1  Hs00252034_m1 |  | *SLC2A2*  *SOX9*  *SREBF1*  *TAT* | Hs01096908_m1  Hs01001343_g1  Hs01088691_m1  Hs00356930_m1 |
| *FAH*  *FASN*  *G6PC*  *GSK3B* | Hs00164611_m1  Hs01005622_m1  Hs006091785_m1  Hs01047719_m1 |  | *TF*  *TTR*  *UGT1A1*  *rRNA, 18S* | Hs01067777_m1  Hs0174914_m1  Hs02511055_s1  Hs99999901_s1 |

*ACAN, aggrecan; ADH1B, alcohol dehydrogenase 1B; ADH1C, alcohol dehydrogenase 1C; AFP, alpha fetoprotein; ALB, albumin; ARG1, arginase 1; ASL, argininosuccinate lyase; ASS1, argininosuccinate synthase 1; ATP7B, ATPase copper transporting beta; ABCB1, ATP binding cassette subfamily B member 1; ABCB11, ATP binding cassette subfamily B member 11; ABCC2, ATP binding cassette subfamily C member 2; BGLAP, bone gamma-carboxyglutamate protein; CPS1, carbamoyl-phosphate synthase 1; CYP1A1: cytochrome P450 1 subfamily A member 1; CYP3A4: cytochrome P450 3 subfamily A member 4; F7, factor VII; F8, factor VIII; FAH, fumarylacetoacetate hydrolase gene; FASN, fatty acid synthase; GSK3B, glycogen synthase kinase 3 beta gene; HNF1A, hepatocyte nuclear factor 1A; HNF4A, hepatocyte nuclear factor 4A; HNF6, hepatocyte nuclear factor 6; KRT18, keratin 18; KRT19, keratin 19; KRT7, keratin 7 gene; LPL, lipoprotein lipase; MME, membrane metalloendopeptidase; NAGS, N-acetylglutamate synthase; NR1I2, nuclear receptor subfamily 1 group I member 2; OTC, ornithine transcarbamylase; PPARA, peroxisome proliferator activated receptor alpha; PPARG, peroxisome proliferator-activated receptor gamma; PROS, protein S; RUNX2, runt related transcription factor 2; SLC2A2, solute carrier family 2 member 2; SOX9, SRY-box9; SREBF1, sterol regulatory element binding transcriptional factor 1; TAT, tyrosine aminotransferase; TF, transferrin gene; TR, and transthyretin; UGT1A1, uridine 5'-diphospho (UDP)-glucuronosyltransferase 1A1.*

**Supplementary Table 4.** List of TaqMan probes for mouse genes.

| **Gene names** | **Gene assay ID Numbers** |
| --- | --- |
| *Acta2* | Mm00725412_s1 |
| *Col1a1* | Mm00801666_g1 |
| *Mmp2* | Mm00439498_m1 |
| *Tgfb1* | Mm01178820_m1 |
| *Tnfa* | Mm00443258_m1 |
| 18S rRNA | Mm03928990_g1 |

*Actaa2, actin, alpha 2, smooth muscle; Col1a1, collagen, type I, alpha 1; Mmp2, matrix metalloprotease 2, Tgfb, transforming growth factor beta; Tnfa, tumor necrosis factor alpha.*
